# Co-Cation Engineering via Mixing of Acetamidinium and Rubidium in FASnI_3_ for Tin Perovskite Solar Cells to Attain 14.5% Efficiency

**DOI:** 10.1021/acs.jpclett.4c01695

**Published:** 2024-07-24

**Authors:** Chun-Hsiao Kuan, Tzu-Shen Liao, Sudhakar Narra, Yi-Wei Tsai, Jhih-Min Lin, Guan-Ruei Chen, Eric Wei-Guang Diau

**Affiliations:** † Department of Applied Chemistry and Institute of Molecular Science, National Yang Ming Chiao Tung University, 1001 Ta-Hseuh Road, Hsinchu 300093, Taiwan; ‡ Center for Emergent Functional Matter Science, National Yang Ming Chiao Tung University, 1001 Ta-Hseuh Road, Hsinchu 300093, Taiwan; § National Synchrotron Radiation Research Center, 101 Hsin-Ann Road, Hsinchu Science Park, Hsinchu 30076, Taiwan

## Abstract

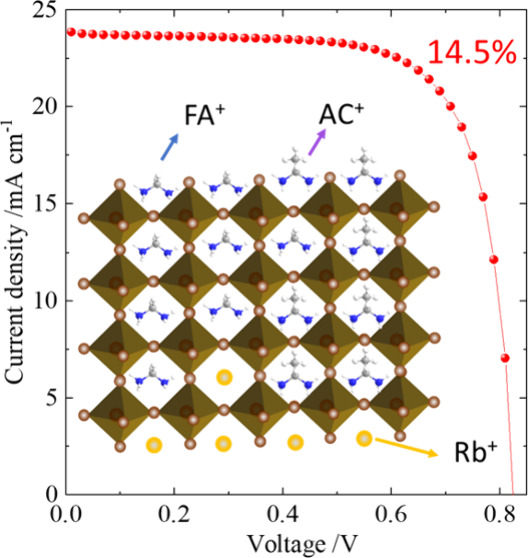

Tin perovskite solar cells (TPSCs) were developed by adding the co-cations acetamidinium (AC) and rubidium (Rb) in varied proportions based on the FASnI_3_ structure (E1). We found that adding 10% AC and 3% Rb can optimize the device (E1AC10Rb3) to attain an efficiency of power conversion of 14.5% with great shelf- and light-soaking stability. The films at varied AC and Rb proportions were characterized using XPS, SEM, AFM, GIWAXS, XRD, TOPAS, TOF-SIMS, UV–vis, PL, TCSPC, and femtosecond TAS techniques to show the excellent optoelectronic properties of the E1AC10Rb3 film in comparison to those of the other films. AC was found to have the effect of passivating the vacancy defects on the surface and near the bottom of the film, while Rb plays a pivotal role in passivating the bottom interface between perovskite and PEDOT:PSS. Therefore, the E1AC10Rb3 device with a band gap of 1.43 eV becomes a promising candidate as a narrow band gap device for tandem lead-free perovskite solar cell development.

Tin perovskite solar cells (TPSCs) have emerged as a leading contender in the popular field of lead-free perovskite solar cells, marking significant progress in next-generation photovoltaic technologies.^1^ However, TPSC faces inherent challenges such as Sn^2+^/Sn^4+^ oxidation, rapid crystallization, and limited stability, which require resolutions to enhance its device performance.^2−4^ Various strategies have been explored to address these issues and promote the device performance of TPSC with a power conversion efficiency (PCE) exceeding 14%. For example, upon adding an interfacial layer of pyridine-functionalized fullerene between the perovskite and electron transport layer (ETL), Zhu and co-workers reported a PCE of 14.14%.^5^ Wei and co-workers used varied C60 derivatives as the ETL with one of the trans-fullerene derivatives attaining a PCE of 14.58%.^6^ Ning and co-workers used the method of one-step synthesis of SnI_2_(DMSO)_*x*_ adducts for TPSC to attain a PCE of 14.6%;^7^ the same group also used PEABr-PEASCN additives to form the 2D–3D structure for TPSC to reach a PCE of 14.6%.^8^ Quasi-2D tin perovskite solar cells offer a significant advancement in photovoltaic technology by combining the benefits of 2D and 3D perovskites. This hybrid structure enhances stability and efficiency, addressing the inherent challenges of tin-based perovskites. The quasi-2D architecture improves moisture and thermal resistance while optimizing charge carrier dynamics through quantum confinement effects and reduced dimensionality. Recent studies have demonstrated the potential of quasi-2D tin perovskite solar cells to achieve high performance with improved longevity.^9,10^ Zhou and co-workers used the approach of chemothermal surface dedoping to remove the oxidized SnI_4_ via formamidinium (FA) chloride (FACl) for TPSC to obtain a PCE of 14.7%.^11^ He and co-workers used FPEABr to form a 2D capping layer on the 3D tin perovskite for TPSC to attain a PCE of 14.81%.^12^ Recently, Wei and co-workers used isomeric fulleropyrrolidines as additives for TPSC to reach a PCE of 15.38%.^13^ Mi and co-workers used FPEABr to form interfacial dipoles to attain a PCE of 15.7%,^15^ which is a record efficiency among all of the TPSCs reported elsewhere. Therefore, additive engineering is a promising approach to effectively promoting the performance of TPSC.^15−19^ This approach helps in passivating surface defects, reducing Sn^4+^ back to Sn^2+^, controlling crystallization, and forming surface-protected low-dimensional perovskites.^7,20−24^

Besides the aforementioned approaches, cationic, anionic, and multifunctional additives have been widely considered to solve the instability problem for TPSC to attain great performance.^25^ We have previously focused on additive engineering using various organic cations such as guanidinium (GA),^15^ 2-hydroxyethylammonium (HEA),^26^ aziridinium (AZ),^17^ and imidazolium (IM)^27^ as A-site co-cations to mix with FA to enhance the performance and stability for TPSC. These co-cations had the effect of passivating surface and vacancy defects and preventing tin(II) oxidation for enhanced performance to attain a PCE of 12.5% for the IM/Cs/FA hybrid system.^27^ Therefore, the incorporation of functional organic cations in TPSC is a promising approach to further promoting the device performance and stability for TPSC.^28^

In this study, we introduced acetamidinium (AC), a bifunctional organic cation, to mix with FA, forming a co-cationic tin perovskite for TPSC. Like FA, AC has two nitrogen atoms but one more methyl group (Figure 1) to replace the hydrogen in the central carbon of FA. The two nitrogen atoms offer dual functionality: one nitrogen can form a hydrogen bond with the SnI_6_^4–^ metal halide framework, and the other one can act as a Lewis base to stabilize the undercoordinated Sn atoms. The restricted C–N bond rotation in AC is due to the delocalized π-electron cloud over the N–C–N bond, which strengthens the resulting N–H···I bond, increasing the electrostatic interaction between the AC cation and the SnI_6_^4–^ octahedron. These interactions can stabilize the perovskite matrix, thereby enhancing its stability.^29^ The size of AC is slightly larger than FA,^30^ but its rigid structure helps to modulate the crystal structure of tin perovskite, maintaining a tolerance factor of close to 1. Therefore, AC might occupy the vacancies of FA to passivate the vacancy defects for a tin perovskite. Additionally, we also include an inorganic cation, rubidium (Rb), known for its stability to accommodate FA and AC. Rb was found to have the effect of passivating the bottom interface between the perovskite and the hole-transport layer (HTL). This FA/AC/Rb triple cationic system aims to replicate the success of its lead-based counterpart in terms of performance and stability. As a result, a short-circuit current density (*J*_SC_) of 23.9 mA cm^–2^, an open-circuit voltage (*V*_OC_) of 0.84 V, and a fill factor (FF) of 0.72 were obtained to give a PCE of 14.5% for the champion device, which is among the top level of efficiencies for TPSC based on the simple perovskite structure, FASnI_3_. Figure 1 illustrates the chemical structure of a triple cationic tin perovskite containing FA, AC, and Cs that is crucial to achieving the optimal performance for TPSC reported herein.

**Figure 1:**
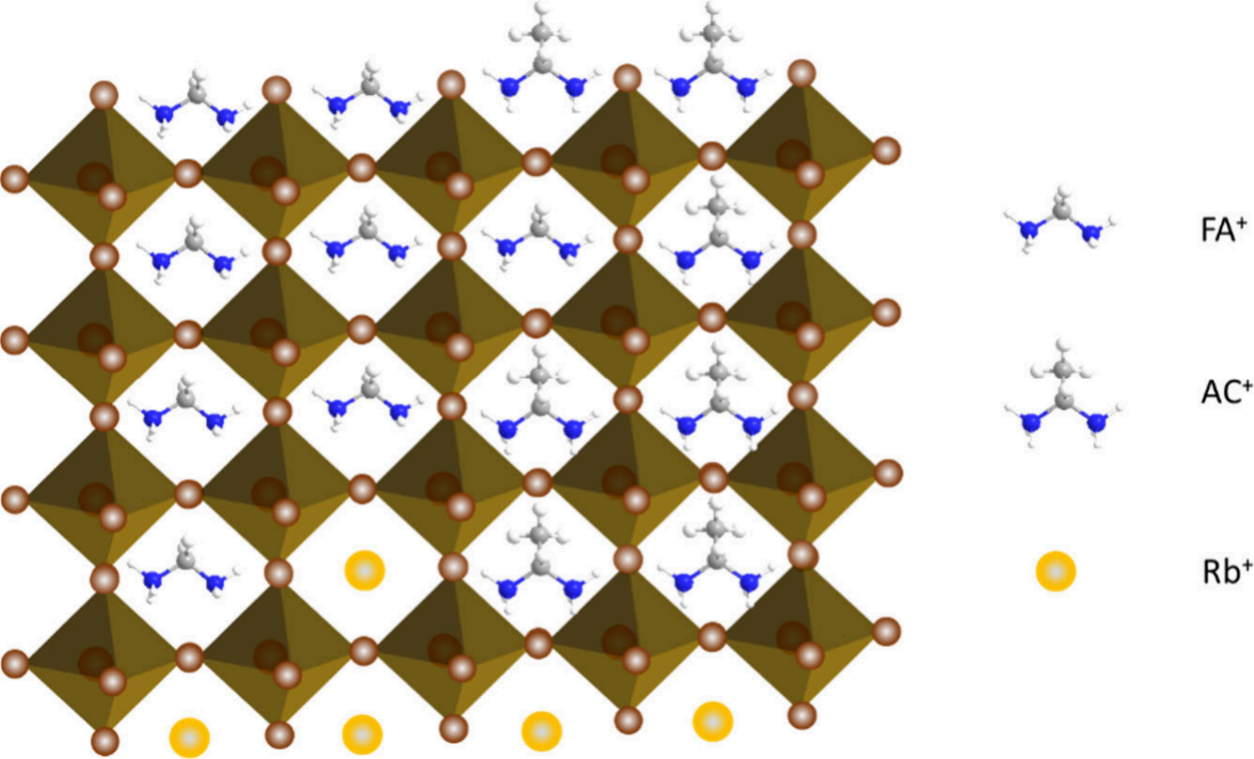
Schematic demonstration of the chemical structure for the tin perovskite material E1AC10Rb3.

To build the best triple cationic tin perovskite system, we started with FASnI_3_ with additives of 10% SnF_2_ and 1% EDAI_2_ (E1) by adding AC and Rb step by step in a systematic way. First, the AC device was optimized with the addition of AC in E1 from 5 to 20% (Figure S1); the best performance occurred at 10% AC addition (E1AC10). Then, the Rb device was optimized with the addition of Rb in E1AC10 from 1% to 5% (Figure S2); the best performance occurred at 3% Rb addition (E1AC10Rb3). Thereafter, we characterized the perovskite films based on E1, E1AC10, and E1AC10Rb3 with AC and Rb in varied proportions.

The X-ray photoelectron spectra (XPS) of these samples are shown in Figure 2a–c; they were analyzed and divided into two distinct parts, representing Sn^2+^ (indicated in red) and Sn^4+^ (in green). The respective proportions of Sn^2+^ and Sn^4+^ are tabulated in Table S1 in the Supporting Information. These findings imply that adding AC in a small quantity aids in improving the stability of the tin perovskite by limiting the surface oxidation of Sn^2+^ to some extent, but the Sn^2+^/Sn^4+^ ratio can be significantly reduced by introducing both AC and Rb together in the E1AC10Rb3 sample. Note that the addition of Rb made a big improvement in preventing Sn^2+^ oxidation; its role inside the perovskite will be discussed in a later section. Figure 2d–f,g–i displays atomic force microscopy (AFM) and scanning electron microscopy (SEM) images, respectively, for the three representative samples. The SEM images reveal good morphologies for all films, but with the E1AC10Rb3 film exhibiting the largest grain sizes compared to the others. Although the E1AC10 also shows good crystal morphology (Figure 2h), the surface roughness (RMS 37.6 nm) is larger than that of the E1 film (RMS 30.1 nm). In contrast, the E1AC10Rb3 film demonstrates an average roughness of RMS 18 nm, which is much smaller than those of E1 and E1AC10, consistent with the corresponding SEM images showing surface morphologies with the same trend. Figures S3 and S4 present the SEM images for the E1ACx and E1AC10Rby samples with x and y in varied proportions. For the AC samples, the SEM images with AC proportions greater than 15% show poor morphology; for the Rb samples, we found that the grain sizes increase from 1 to 3% and then decrease from 5 to 98% with poor morphologies for high proportion samples showing the pin holes between the crystal grains. The AFM images (Figures S5 and S6) exhibit large variations in film roughness for the AC samples, but a systematic trend was observed for the Rb samples, with the 3% sample (E1AC10Rb3) showing the best film roughness compared to the others. To examine the crystallinity and phase stability, we carried out grazing incidence wide-angle X-ray scattering (GIWAXS) experiments at the TPS 25A1 beamline facility located at the National Synchrotron Radiation Research Centre (NSRRC) in Taiwan. These tests were conducted on perovskite films made of E1, E1AC10, and E1AC10Rb3. The findings from the GIWAXS measurements are illustrated in Figure 2j–l. For the GIWAXS patterns, a significant observation was to find the phase transition from cubic to SnI_2_ for both E1 and E1AC10 films, while the E1AC10Rb3 film retains phase purity with great crystallinity.

**Figure 2:**
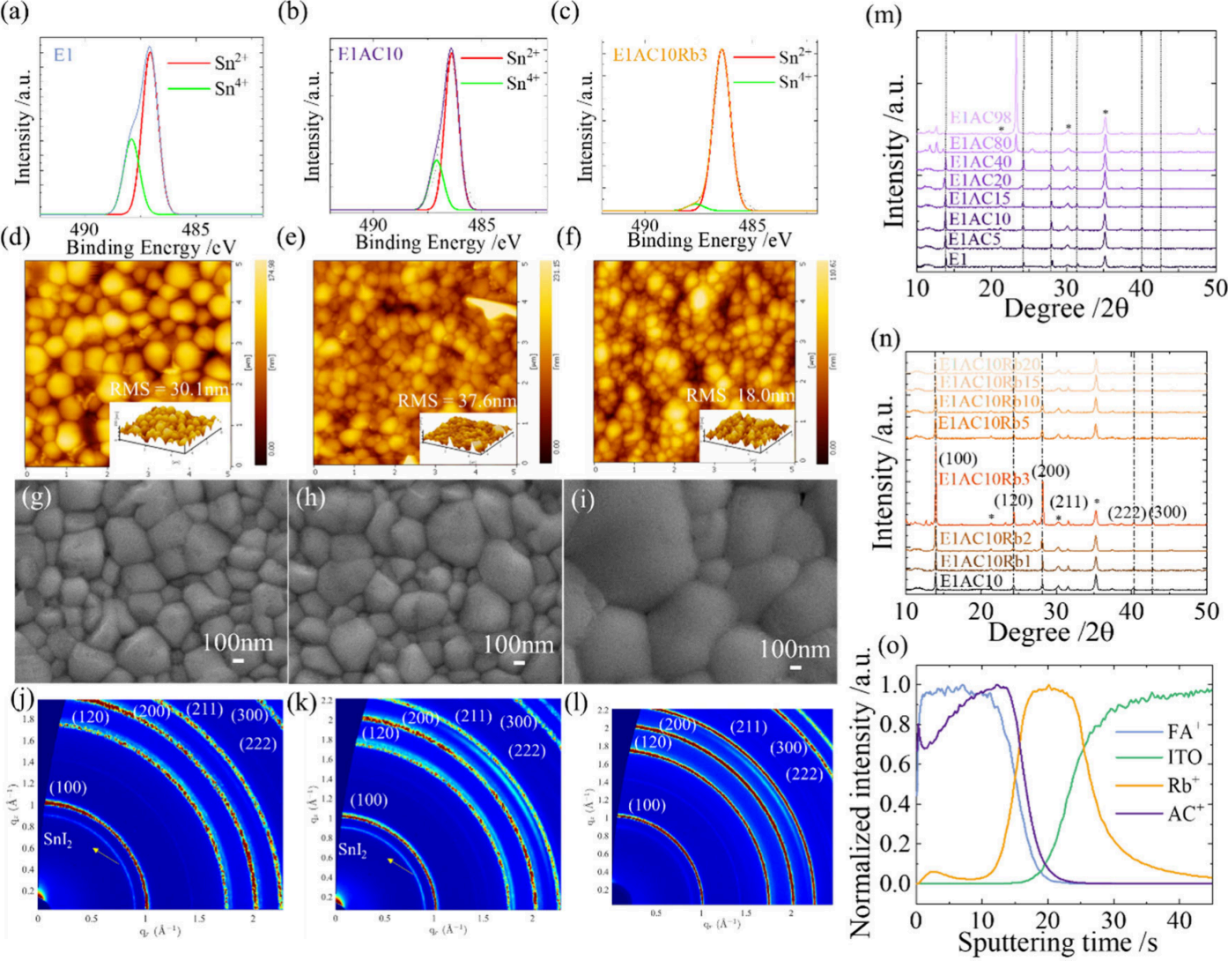
Characterization of thin-film samples for the XPS spectra of (a) E1, (b) E1AC10, and (c) E1AC10Rb3 showing the Sn^2+^/Sn^4+^ proportions. (d–f) AFM and (g–i) SEM images of E1, E1AC10, and E1AC10Rb3 showing the morphological character. (j–l) GIWAXS patterns showing the corresponding 2D crystallinity patterns for E1, E1AC10, and E1AC10Rb3. XRD patterns of the different ratios of (m) ACx (x = 0–98) and (n) Rby (y = 0–20). (o) TOF-SIMS distribution plots of the E1AC10Rb3 sample.

The X-ray diffraction (XRD) patterns for the AC and Rb samples are shown in Figure 2m,n, respectively, in varied AC and Rb proportions. For the AC samples, a noticeable shift toward lower diffraction angles in the large AC proportions was observed when compared to the E1 film, as illustrated in Figure 2m. The corresponding total pattern solution (TOPAS) fits of the XRD patterns are shown in Figures S7–S12, and the fitted lattice parameters are tabulated in Table S2. Because AC is larger than FA, the incorporation of AC into E1 increases the size of the lattice in a pure cubic phase (Table S2). For the Rb samples, due to the small size of Rb, incorporating Rb slightly decreases the size of the lattice, but the effect of the size decrease was not apparent when the proportion of Rb increased (Figures S13–1S9 and Table S3). Furthermore, the XRD peak intensities were observed to increase upon increasing the amounts of AC and Rb to 10 and 3%, respectively, and then to decrease thereafter. As a result, the E1AC10Rb3 film demonstrated the highest level of crystallinity among all of the films. We clearly identified diffraction patterns corresponding to the (100), (120), (200), (211), (222), and (300) facets, with the expected cubic phase of the crystals for all samples.

Time-of-flight secondary ion mass spectrometry (TOF-SIMS) was applied to study the lateral ion distribution of the cations inside the E1AC10Rb3 film (Figure 2o). The TOF-SIMS analysis revealed a smooth lateral distribution of the FA cation but with vacancy defects on the surface and in the bottom region of the perovskite film. The AC cation, marked by its two N–H bonds in a rigid structure with an additional methyl group, appears to be instrumental in stabilizing tin atoms that are undercoordinated and involve related charge-trapping defect states. Additionally, these N–H bonds in AC can form hydrogen bonds with iodine atoms located at the grain boundaries to passivate the surface defects caused by missing FA in the surface region. Furthermore, the FA vacancy defects existed not only on the surface of the perovskite but also at the perovskite/HTL interface. As shown in Figure 2o, AC had some effects of passivating the FA vacancy defects in the bottom of the film as well as in the bulk. More importantly, the TOF-SIMS results indicate that the lateral distribution of Rb mostly occupies the bottom of the film, implying that Rb plays an essential role in effectively mitigating the interfacial defects, thus contributing to the enhanced performance of the devices.

For optical characterization of the hybrid perovskite films, we show the UV–visible absorption spectra, photoluminescence (PL) spectra, and PL decay profiles of the AC samples in Figure 3a–c, respectively, while those of the Rb samples are in Figure 3e–f, respectively. Incorporation of the AC cation leads to a significant blue spectral shift in both absorption and PL spectra. This makes a widening of the band gap (Eg) starting from the E1 sample (1.36 eV) and increasing to the E1AC98 sample (2.29 eV), with the E1AC10 sample having an Eg of 1.42 eV. Staring from E1AC10, Eg continues to increase when the Rb cation is included, but the extent of the increase was small. For the optimal E1AC10Rb3 sample, Eg is 1.43 eV. The PL decay profiles (Figure 3c,f) were obtained from time-correlated single-photon-counting (TCSPC) measurements at an excitation wavelength of 635 nm, and the signals were collected at the peak position of the corresponding PL spectra. These PL decay profiles were fitted using a bi- or triexponential function, with the fitted parameters listed in Tables S4 and S5. The decays of PL profiles are due to both radiative and nonradiative processes in the lowest excited state. The rapid decays thus indicate an efficient nonradiative process to compete with the radiative process. For the present case under investigation, rapid decays should be due to the defect states that trap the charges as an effective nonradiative process. Therefore, a longer PL lifetime would mean fewer defect states inside the film. Consequently, we observed the AC samples to have the longest PL lifetime at E1AC10 (11.8 ns) and Rb at E1AC10Rb3 (21.2 ns). This is consistent with our device performance for the E1AC10Rb3 device having the best performance due to fewer defect states being produced after surface and interfacial passivation via AC and Rb.

**Figure 3:**
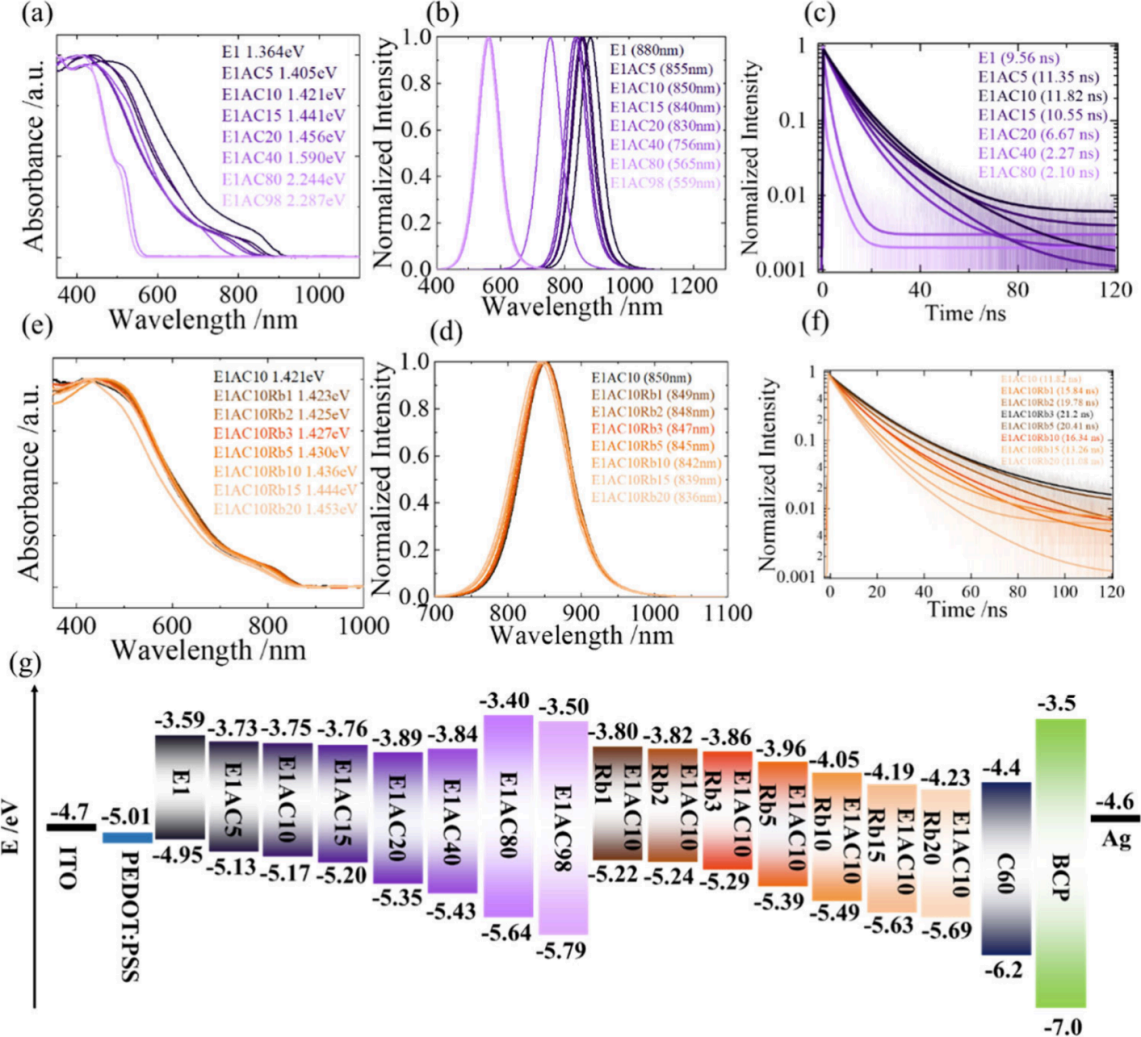
(a, e) UV–vis, (b, d) PL, and (c, f) TCSPC decays of E1ACx and E1AC10Rby with different ratios as indicated. (g) Energy-level diagram of the E1ACxRby films as indicated.

Ultraviolet photoelectron spectroscopy (UPS) measurements were conducted for each AC and Rb sample, with the results shown in Figures S20–S23. The energy levels of the valence band maximum (VBM) were determined by the UPS data, and those of the conduction band minimum (CBM) were determined by raising the energy levels with the corresponding band gaps determined previously. Figure 3g shows the energy-level diagram for each sample under investigation to compare with other components required to compose a TPSC. In all instances, the VBM levels are suitable for hole transfer to the HOMO level of PEDOT:PSS, and the CBM levels are conducive to electron transfer to the LUMO level of C60.

Femtosecond transient absorption spectral (TAS) contour plots of the E1, E1AC10, and E1AC10Rb3 samples are shown in Figure 4a–c, respectively. The TAS profiles show signals corresponding to their respective ground-state photobleaches (PB) and photoinduced absorption bands (PIA) as shown in Figure 4d. The E1AC10Rb3 sample showed stronger TAS intensities at longer time scales on the spectrograms (Figure 4a–c) compared with those of the E1 and E1AC10 samples. Femtosecond TAS profiles show PB bands associated with the band edges, referred to as the PB1 band, at 854, 820, and 815 nm for E1, E1AC10, and E1AC10Rb3 samples, respectively. The PB1 band position of E1 is consistent with the work reported elsewhere.^31,32^ The blue shifts of the PB1 band upon the incorporation of additional AC and Rb cations could be due to the distortion caused to the lattice by the change in the cation environment from FA to FA + AC and FA + AC + Rb, respectively. In our earlier works, we have shown that the addition of additives induces a blue shift to the band edges of tin perovskites.^15−17^ Similar to the PB1 band, the high-energy PB band, referred to as the PB2 band, also shows a blue shift of about 20 nm from E1 to E1AC10Rb3 samples. Interestingly, the spacing between the energy levels was also reduced upon the addition of AC and Rb cations. Notably, the PB2 band was shown to be that of splitting caused by spin–orbit coupling effects in tin perovskite nanocrystal samples.^33^ The transient kinetics of the PB1 bands were monitored to probe the thermalization and trap-state-mediated recombination, as shown in Figure 4e,f. Figure 4f shows that thermalization time coefficients in the rising feature slowed from 0.3 to 0.5 to 1.5 ps upon adding of additives AC and AC + Rb, due to the suppression of hot carrier cooling rates via these cations. The slowdown of hot carrier cooling rates can be rationalized based on the modulation of phonon modes by newly incorporated cations Rb and AC.^34,35^ Additionally, these additives also suppressed the picosecond trapping channels seen in E1 (7 ps) to a great extent in the E1AC10Rb3 sample (no such relaxation component was observed), which could lead to better charge extraction for the latter than for the former.

**Figure 4:**
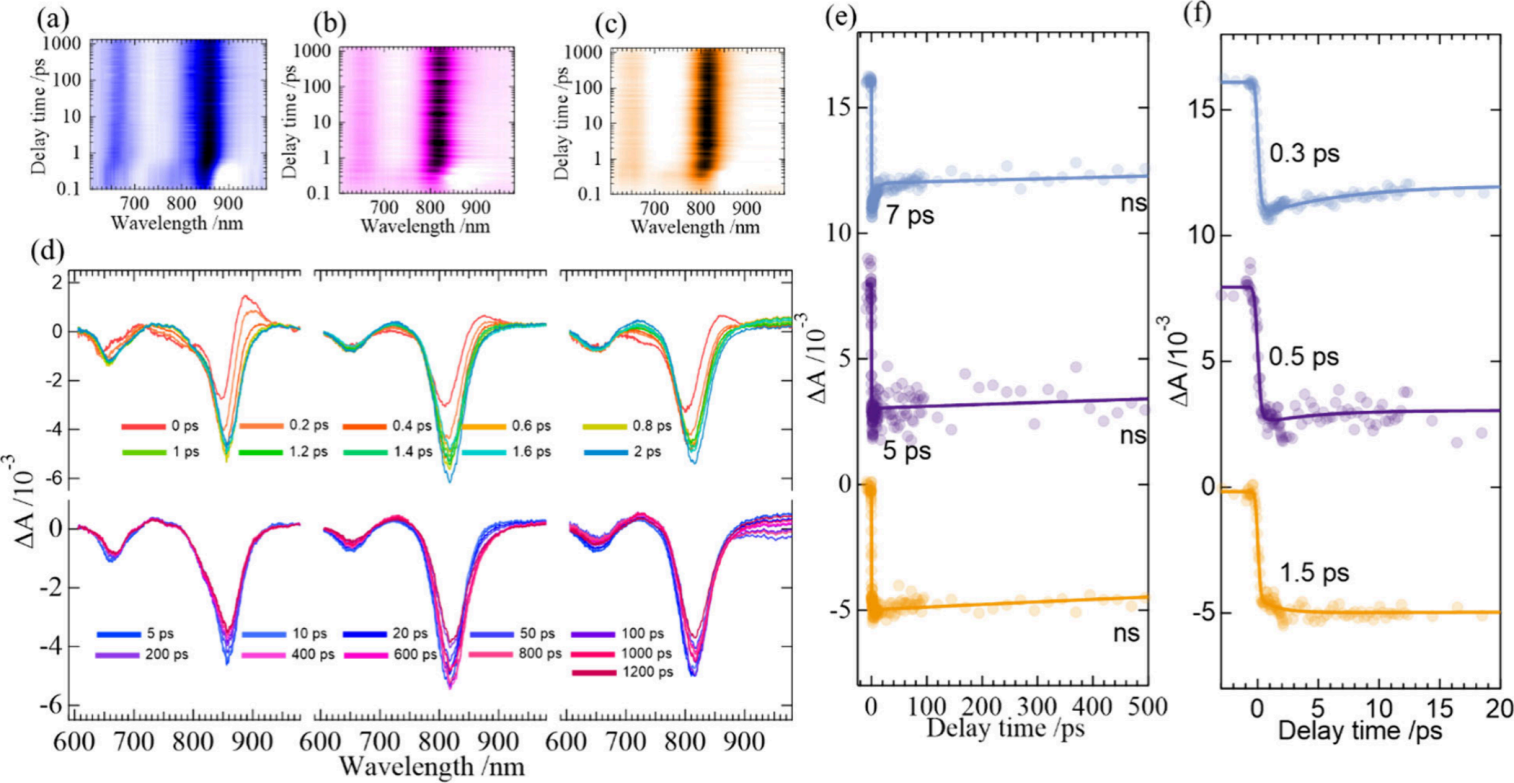
Femtosecond transient absorption spectrograms of FASnI_3_ for the samples of (a) E1, (b) E1AC10, and (c) E1AC10Rb3. (d) Corresponding femtosecond transient absorption spectra showing the temporal TAS profiles for E1, E1AC10, and E1AC10Rb3 samples. (e, f) TAS kinetics of PB1 bands of the E1, E1AC10, and E1AC10Rbs samples showing the recovery transient profiles in long- and short-time regions, respectively.

The devices were fabricated following the inverted cell structure ITO/PEDOT:PSS/perovskite/C60/BCP/Ag. Figure 5a presents the current–voltage (*J*–*V*) characteristics of the best devices made of perovskites E1, E1AC10, and E1AC10Rb3. The PCE of the best E1 device was relatively low at 8.8%, which is consistent with our previous reports.^14^ With the addition of AC^+^, the PCE of the best E1AC10 device slightly increased to 10.3%. However, a significant improvement was observed when both AC^+^ and Rb^+^ were incorporated into E1, with the best E1AC10Rb3 device achieving a remarkable PCE of 14.5% from a forward *J*–*V* scan with little effect of hysteresis (Figure S24). Figure 5b displays the incident photon-to-current conversion efficiency (IPCE) spectra of the three devices, and the integrated current densities from these spectra are in good agreement with those obtained from the *J*–*V* measurements. To understand the charge recombination characteristics of these devices, electrochemical impedance spectroscopy (EIS) studies were conducted. The Nyquist plots of these EIS measurements, performed in the dark at a bias voltage of 0.5 V, are depicted in Figure 5c. The Nyquist plots for all devices showed a single semicircle, analyzed using a simple RC equivalent circuit model. The impedances obtained from this model reveal a trend in the order of E1AC10Rb3 ≫ E1AC10 > E1, suggesting a similar trend in their abilities to inhibit charge recombination. These EIS results are consistent with the observed improvements in *V*_OC_, *J*_SC_, and PCE showing the same order for the E1AC10Rb3 device to outperform the others. Figure S25 shows the reproducibility of the device performance through boxplots for 30 devices of each sample (E1, E1AC10, and E1AC10Rb3), all tested under identical experimental conditions. The photovoltaic parameters for these devices are summarized in Tables S6–S8. Figure 5d illustrates the shelf-storage stability of each device, stored in a glovebox without encapsulation. Notably, the E1AC10Rb3 device maintained over 90% of its initial performance for 3000 h, significantly outperforming the other two devices. Furthermore, the E1AC10Rb3 device was stable under 1 sun illumination at the maximum power point (MPP) for over 7 h (Figure S26). Therefore, the E1AC10Rb3 device exhibited great device stability with a band gap of 1.43 eV suitable for a low-band-gap TPSC for its potential use in future lead-free all-perovskite tandem solar cells.

**Figure 5:**
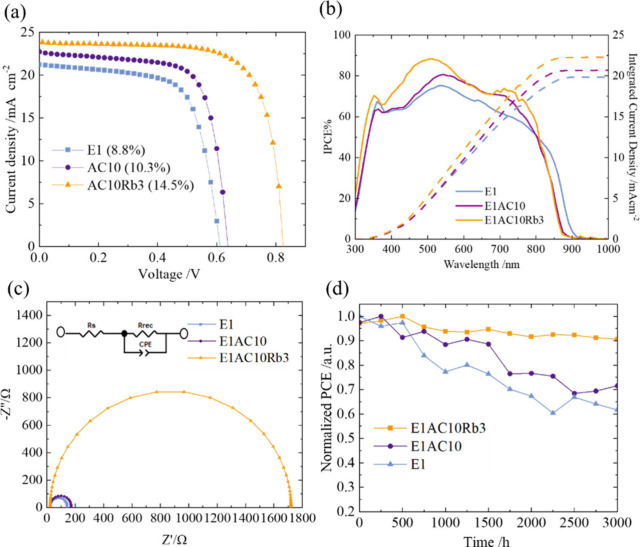
(a) *J*–*V* characteristic curves, (b) IPCE spectra, (c) Nyquist plots from EIS measurements, and (d) normalized plots of PCE vs the shelf-storage period showing the long-term stability of the E1, E1AC10, and E1AC10Rb3 devices.

In conclusion, we developed a mixed co-cationic tin perovskite solar cell based on the structure of FASnI_3_ (E1) with the addition of acetamidinium (AC) and rubidium (Rb) in a systematic way. The device was optimized with varied proportions of AC based on the E1 device, and it was found that the best condition occurred at 10% AC (E1AC10). Then the device was optimized with varied proportions of Rb, and it was found that the best condition occurred at 3% Rb (E1AC10Rb3). The AC and Rb films in varied proportions were characterized using the techniques of XPS, SEM, AFM, GIWAXS, XRD, TOPAS, TOF-SIMS, UV–vis, PL, TCSPC, and femtosecond TAS. The E1AC10Rb3 film was found to show a larger Sn^2+^/Sn^4+^ ratio, greater surface morphology with larger crystal grain size, smaller surface roughness, better phase purity, greater crystallinity, a longer PL lifetime, and a longer hot-carrier cooling time in comparison with the other films. In addition, AC plays a crucial role in passivating the defects on the surface, at the bottom, and in the bulk of the film, whereas Rb plays a key role in passivating the defects between perovskite and PEDOT:PSS. As a result, the best E1AC10Rb3 device exhibited a remarkable PCE of 14.5%, great shelf stability for 3000 h, and excellent light-exposure stability for illumination under ambient 1 sun conditions for 7.5 h at the maximum power point (MPP). With a band gap of 1.43 eV, the E1AC10Rb3 device becomes a promising candidate for future lead-free tandem perovskite solar cell development.

## Supporting Information Available

Experimental methods; supplementary figures and tables for *J*–*V* curves; SEM; AFM; TOPAS; UPS; performance statistics; MPPT; and summary of the device performance (PDF)
Transparent Peer Review report available (PDF)
