# Supplementary material for: Co-Cation Engineering via Mixing of Acetamidinium and Rubidium in FASnI3 for Tin Perovskite Solar Cells to Attain 14.5% Efficiency
Source: J Phys Chem Lett. 2024 Jul 24;15(30):7763–9. doi: 10.1021/acs.jpclett.4c01695 (PMC11299185; doi:10.1021/acs.jpclett.4c01695)
Supplement: Supplementary file 1 — jz4c01695_si_001.pdf [file jz4c01695_si_001.pdf]

Co-Cation Engineering via Mixing of Acetamidinium and Rubidium in FASnI3 for Tin Perovskite Solar Cells to Attain Efficiency 14.5%

Chun-Hsiao Kuan,[a] Tzu-Shen Liao,[a] Sudhakar Narra,[a] Yi-Wei Tsai,[c] Jhih-Min Lin,[c] Guan-Ruei Chen,[c] and Eric Wei-Guang Diau[*,a,b]

[a]Department of Applied Chemistry and Institute of Molecular Science, National Yang Ming Chiao Tung University, 1001 Ta-Hseuh Rd., Hsinchu 300093, Taiwan.

[b]Center for Emergent Functional Matter Science, National Yang Ming Chiao Tung University, 1001 Ta-Hseuh Rd., Hsinchu 300093, Taiwan; E-mail: diau@nycu.edu.tw

[c]National Synchrotron Radiation Research Center, 101 Hsin-Ann Road, Hsinchu Science Park, Hsinchu 30076, Taiwan

Experiments

Chemicals: FAI and ACI (Greatcell Solar),RbI_2 (Sigma- Aldrich), SnI_2 (99.999 %, Sigma- Aldrich), SnF_2 (99 %, Sigma- Aldrich), DMSO (Sigma- Aldrich), $EDAI_2$ (Greatcell Solar), and CsI (99.9%, TCI) were used as received.

Device fabrication:
Commercial-grade Indium Tin Oxide (ITO) glass sheets, each with dimensions of 1.9 by 1.9 square centimeters, were subjected to a thorough cleaning process. This cleaning involved the use of Isopropyl Alcohol (IPA), acetone, and distilled water in a sonication bath, lasting for 30 minutes, to ensure purity before usage. Before applying the Hole Transport Material (HTM), these glass sheets were exposed to UV-ozone treatment for 20 minutes. The HTM, specifically PEDOT:PSS, was applied onto the glass via spin-coating at a speed of 5000 revolutions per minute for a duration of 30 seconds, followed by an annealing process at a temperature of 70℃ for 10 minutes. For crafting the E1AC10Rb3 film, the precursor solution was mixed from various components: FAI (62.12mg), ACI (7.9mg), RbI (2.7mg), SnI_2 (158.31mg), $EDAI_2$ (1.34mg), Sulfamic acid (SA) (3.7mg), SnF_2 (6.66mg) in DMSO (500 ul, 0.85M perovskite solution) spin-coated at 5000 rpm. Chlorobenzene (200 μL) was dripped as antisolvent for 60 s of spin coating. It was then annealed at 70 °C for 10 min. An electron-transport layer (C_{60},

thickness ~35 nm) and a hole-blocking layer (BCP, thickness ~5 nm) was deposited using a thermal evaporation machine. Finally, a top metal layer composed of silver (Ag) with a thickness of approximately 100 nanometers was deposited onto the structure through thermal evaporation under a vacuum pressure of about 5×10^{-6} Torr.

***Characterization of films and devices*:**

The photovoltaic characteristics of the devices were determined using a Keithley 2400 meter under standard solar illumination conditions (AM 1.5G, intensity of 100 mW per square centimeter), provided by a solar simulator (XES-40S1, SAN-E1 brand). This process involved calibrating with a silicon solar cell equipped with a KG-5 filter. All photovoltaic measurements, including reverse scans from open-circuit voltage to 0 volt and forward scans from 0 volt to open-circuit voltage, were performed in normal air conditions. During these tests, a metal mask of 0.1 square centimeter area was used to cover the device. The conversion efficiency of photons to electrons (IPCE) was measured with an apparatus comprising a Xenon lamp (A-1010, PTi, 150 watts) and a monochromator (PTi, 1200 grooves per millimeter, optimized for 500 nanometers wavelength). Calibration of the IPCE spectra was conducted using a standard silicon photodiode (S1337-1012BQ, Hamamatsu brand). X-ray diffraction patterns were obtained using a Bruker D8-Advance diffractometer with copper K-alpha radiation. To examine the morphology and structure of the samples, a high-resolution Scanning Electron Microscope (SEM, Hitachi SU8010 model) and an Atomic Force Microscope (AFM, VT SPM model from SII Nanotechnology Inc.) were utilized. The samples were sealed with a glass cover using UV-sensitive adhesive (NOA 68, Norland Products). UV-visible absorption spectra for the perovskite samples were recorded using a Jasco V780 UV-visible spectrophotometer. Photoluminescence (PL) spectra were acquired using a Continuous Wave diode laser (450 nm wavelength, MDL-III-450-100 mW model; with PSU-III-FDA power supply) as the excitation source. Emission spectra were collected in the 550-1100 nm range using a Dongwoo DM150i spectrometer with 600 grooves optimized for 750 nm, and detected with a thermoelectrically-cooled silicon photodiode (Sciencetech Inc. S-025-TE2-H; powered by a PS/TC-1 supply).

***TCSPC measurements*:**

Time-Correlated Single Photon Counting (TCSPC) measurements were conducted utilizing a TCSPC setup (Fluotime 200, Picoquant brand), which used a laser with an excitation wavelength of 635 nanometers. These measurements focused on capturing transient decay profiles at the peak wavelengths observed in the PL spectra. Throughout these experiments, the laser operated at a consistent repetition rate of 25 MHz, and its pulse energy was maintained at 4 microjoules per square centimeter. The collected transient PL profiles were then meticulously analyzed and interpreted using a bi- or tri-exponential function model to understand their decay characteristics.

Grazing-incidence Wide-angle X-ray Scattering (GIWAXS) measurements:

Grazing Incidence Wide Angle X-Ray Scattering (GIWAXS) studies were carried out at the TPS 25A1 beamline station, located at the National Synchrotron Radiation Research Center (NSRRC) in Taiwan. The X-ray beam used in these experiments had a photon energy of 12.37 keV and its dimensions were finely tuned to 5 μm by 5 μm. By setting the incident angle of the X-ray beam to a minimal 0.05 degree, the area of the beam interacting with the samples was restricted to a span of just a few mm. The experiments were designed with a distance of 85 mm between the sample and the detector, allowing for a measurement range (q-range) extending from 0.1 to 5 Angstroms inverse. This setup, combining a low angle of incidence with a small beam footprint, significantly improved the clarity of the film diffraction patterns. These patterns were then precisely recorded using the Eiger X 1M single-photon counting detector, which features a pixel size of 75 μm.

Time-of-flight secondary-ion mass spectroscopy (TOF-SIMS) measurements:

Depth profiles using Time-of-Flight Secondary Ion Mass Spectrometry (TOF-SIMS) were acquired with a PHI TRIFT V nanoTOF system (manufactured by ULVAC-PHI, Japan), utilizing a dual beam technique that combines slicing and viewing. The data collection process involved the use of a pulsed $C60^+$ primary ion beam, set at an energy of 20 kV, with a pulse frequency of 8200 Hertz and a pulse duration of 15 ns, which was instrumental in generating secondary-ion signals. This primary ion beam maintained a current of 0.15 nA (DC). The targeted area for analysis was defined as 50 μm by 50 μm. To accelerate the secondary ions, a 3 kV bias was applied to the sample. During the data acquisition process, both a 10 V electron flood and a 10 V Ar^+ flood were used to neutralize any surface charge. In order to compensate for variations in the primary ion signal, all secondary-ion signals within the depth profiles were normalized against the total ion signals. For the sputtering phase, aimed at removing material from the surface, a 1 kV Ar^+ ion beam with a current of 100 nA (DC) was utilized. The raster area designated for this Ar^+ sputtering beam was 2 mm by 2 mm.

Femtosecond transient absorption spectral (TAS) measurements:

Femtosecond transient absorption spectra were collected with the Excipro transient absorption spectrometer (manufactured by CDP systems), as detailed in previous reports. The process involved exciting the samples with a 532 nm pump pulse, with a duration of approximately 70 fs, and then probing them in the wavelength range of 570 to 980 nm using a white light supercontinuum probe pulse. The laser fluence at the sample location was estimated to be around 4 μJ per square centimeter. To protect the samples from atmospheric elements and laser-induced damage, they were encaspsulated and continuously rotated throughout the measurement process. The data gathered was then refined by correcting for the chirp and reducing noise through the

application of a singular value decomposition algorithm.

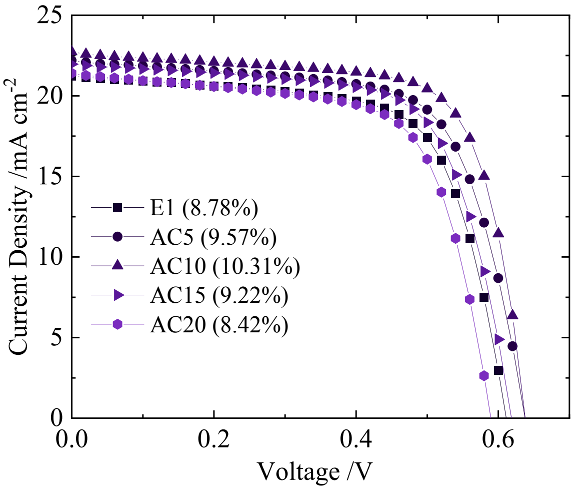

Figure S1. *J-V* measurements of TPSC devices made on different ratios of AC.

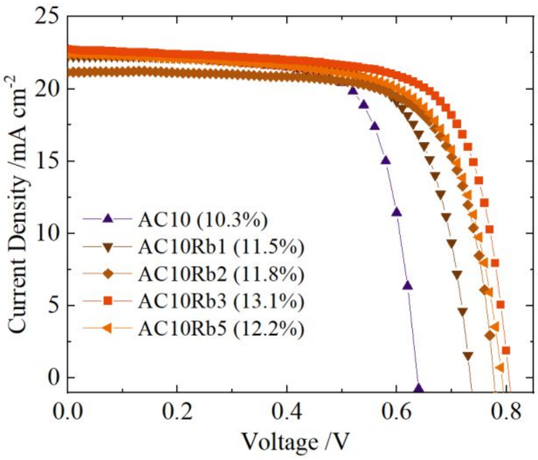

Figure S2. *J-V* measurements of TPSC devices made on different ratios of Rb.

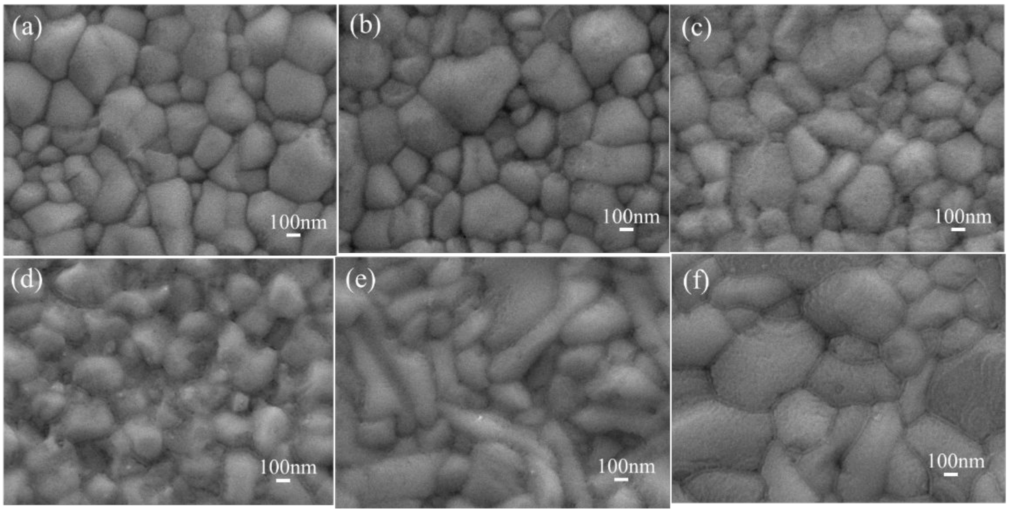

Figure S3. Top-view SEM images of the samples of (a) E1AC5, (b) E1AC15, (c) E1AC20, (d) E1AC40, (e) E1AC80 and (f) E1AC98.
‘

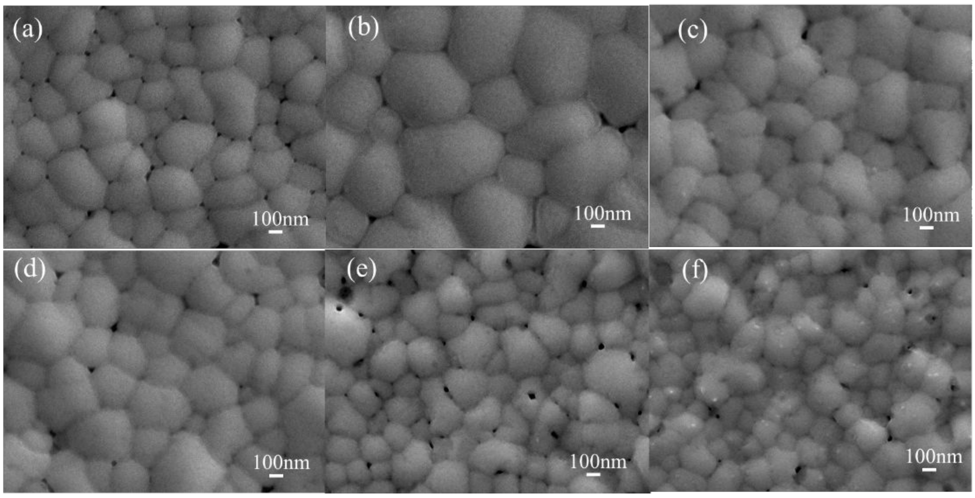

Figure S4. Top-view SEM images of the samples of (a) E1AC10Rb1, (b) E1AC10Rb2, (c) E1AC10Rb5, (d) E1AC10Rb10, (e) E1AC10Rb15 and (f) E1AC10Rb20.

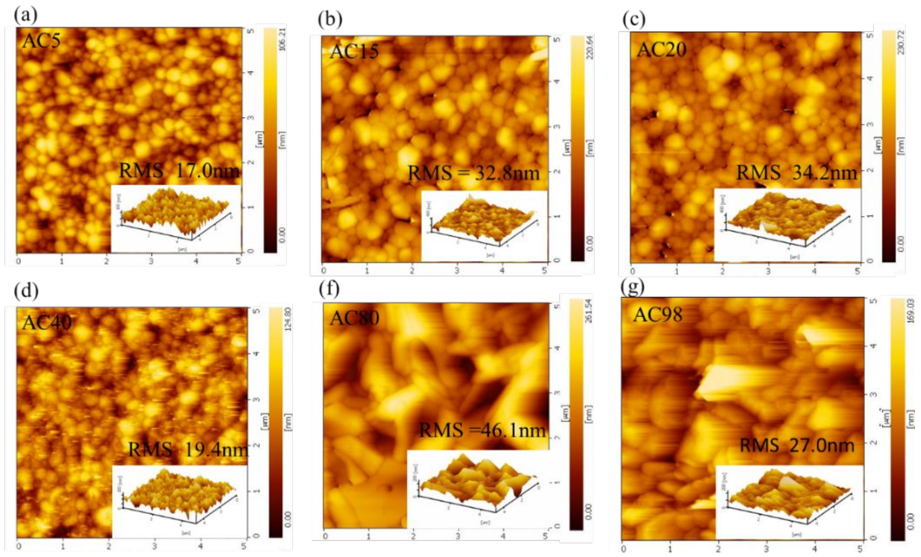

Figure S5. Top-view and 3D (in insets) AFM images of the samples of (a) E1AC5, (b) E1AC15, (c) E1AC20, (d) E1AC40, (e) E1AC80 and (f) E1AC98.

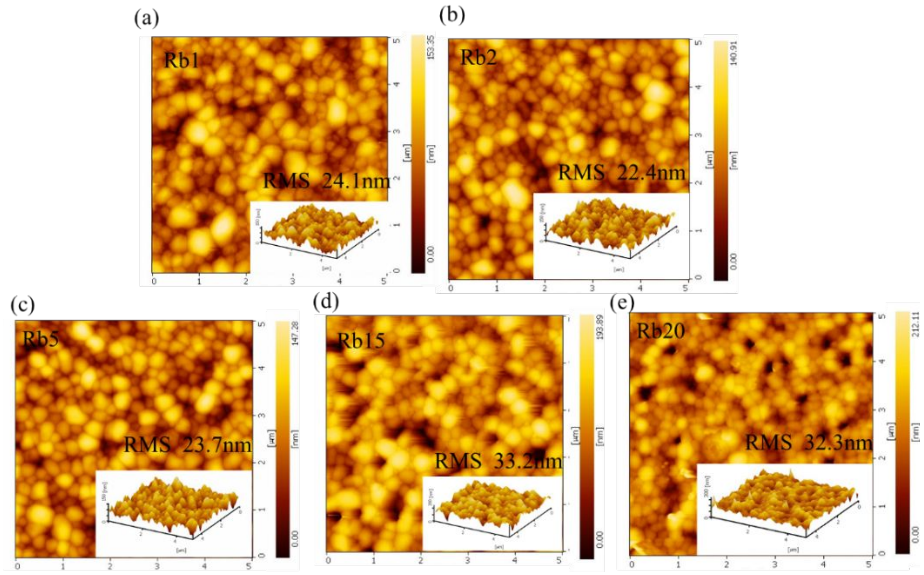

Figure S6. Top-view and 3D (in insets) AFM images of the samples of (a) E1AC10Rb1, (b) E1AC10Rb2, (c) E1AC10Rb5, (d) E1AC10Rb15 and (e) E1AC10Rb20.

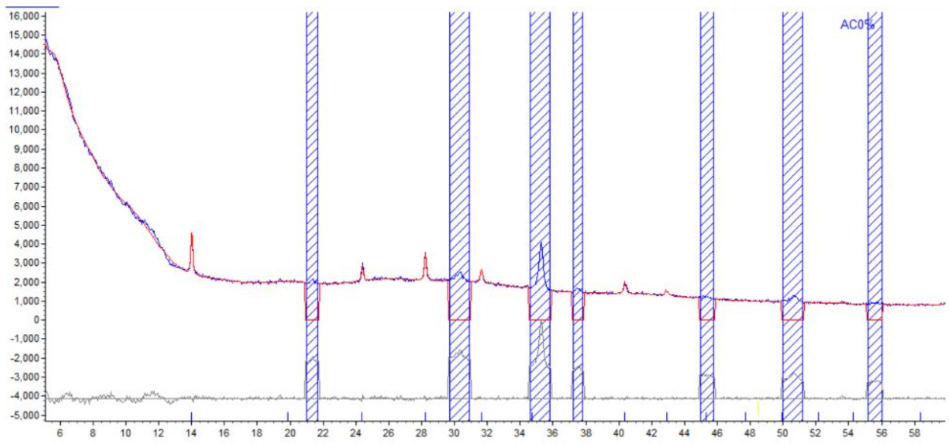

Figure S7. TOPAS fitting of the E1 sample. The shadow area indicates the signals from ITO substrate.

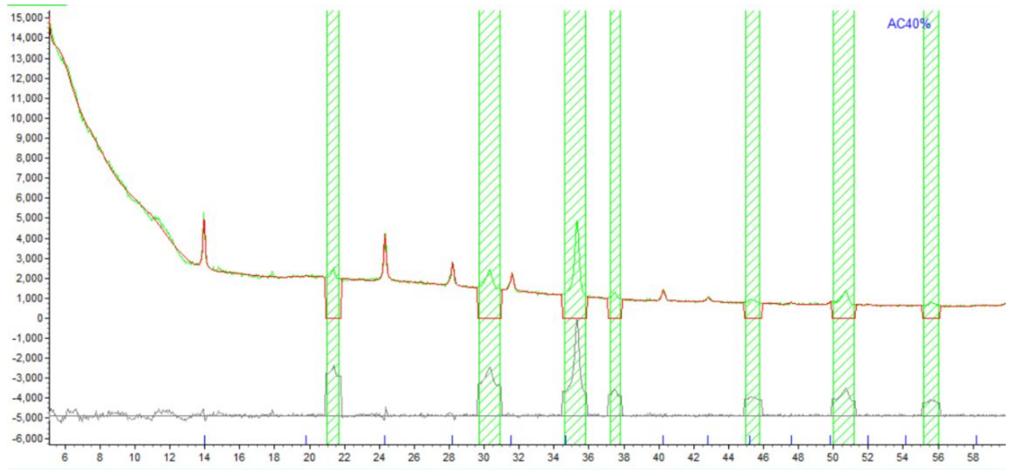

Figure S8. TOPAS fitting of the AC5 sample.

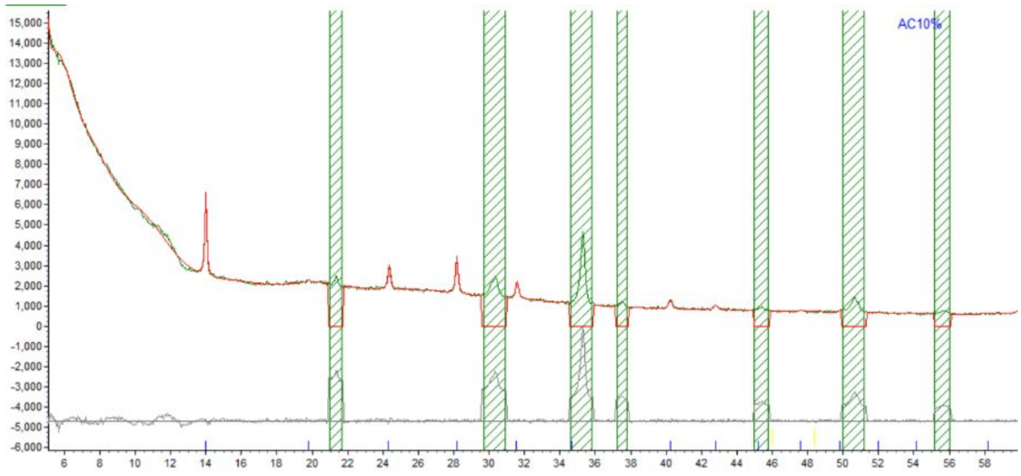

Figure S9. TOPAS fitting of the AC10 sample.

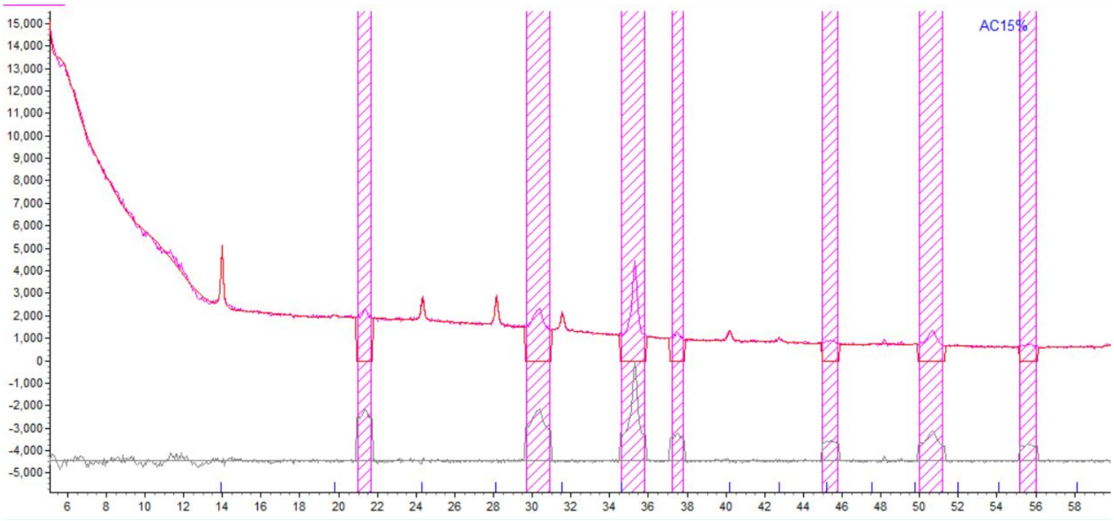

Figure S10. TOPAS fitting of the AC15 sample.

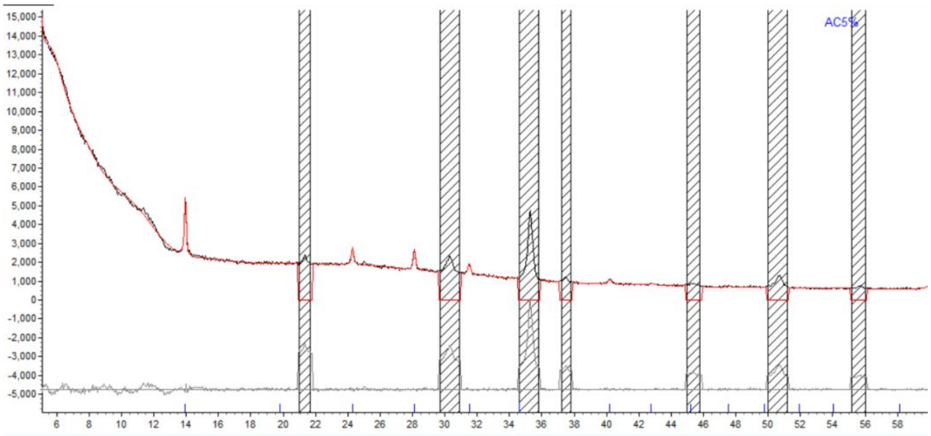

Figure S11. TOPAS fitting of the AC20 sample.

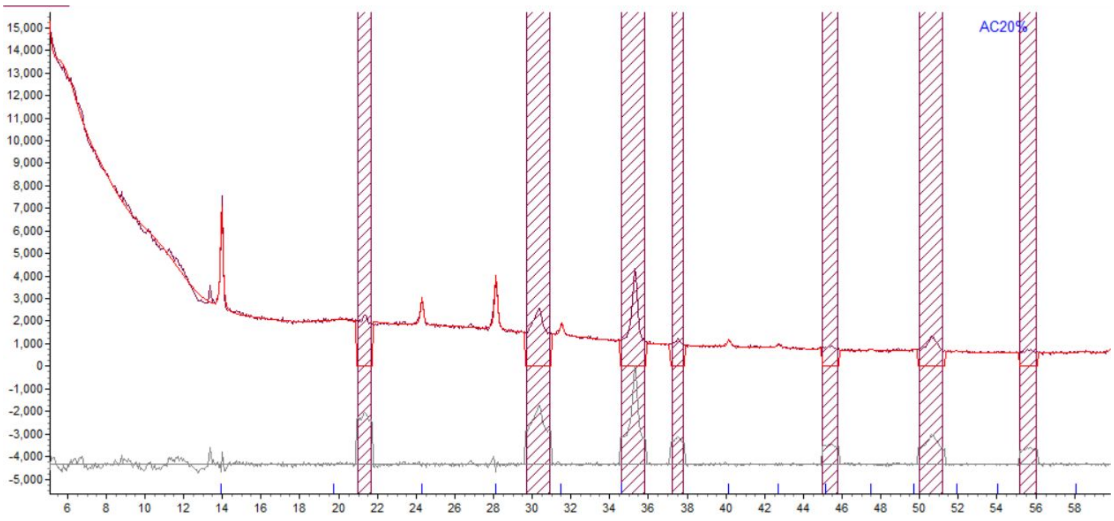

Figure S12. TOPAS fitting of the AC40 sample.

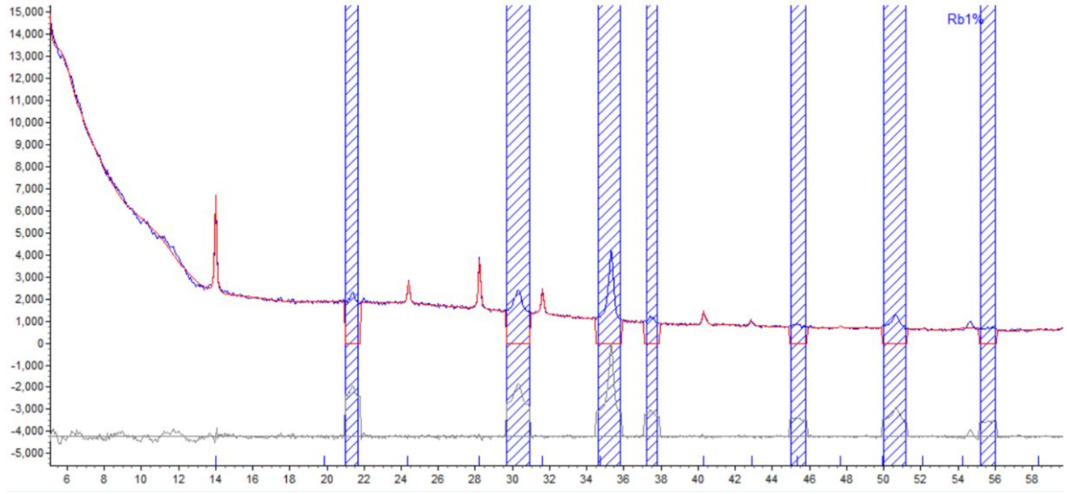

Figure S13. TOPAS fitting of the Rb1 sample.

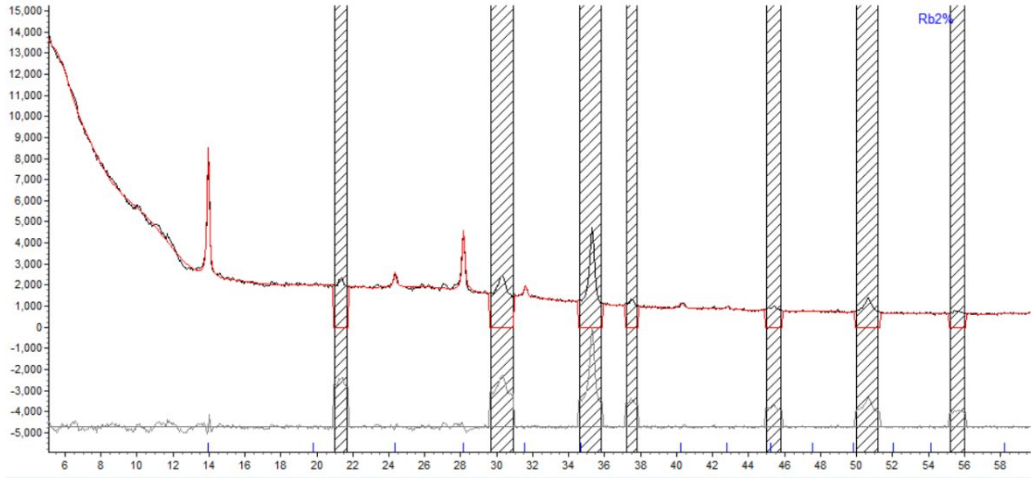

Figure S14. TOPAS fitting of the Rb2 sample.

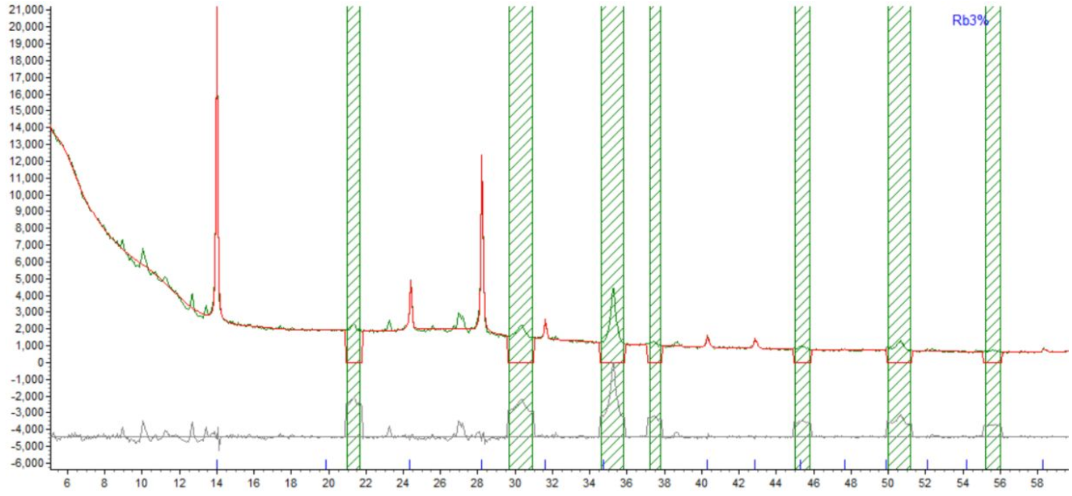

Figure S15. TOPAS fitting of the Rb3 sample.

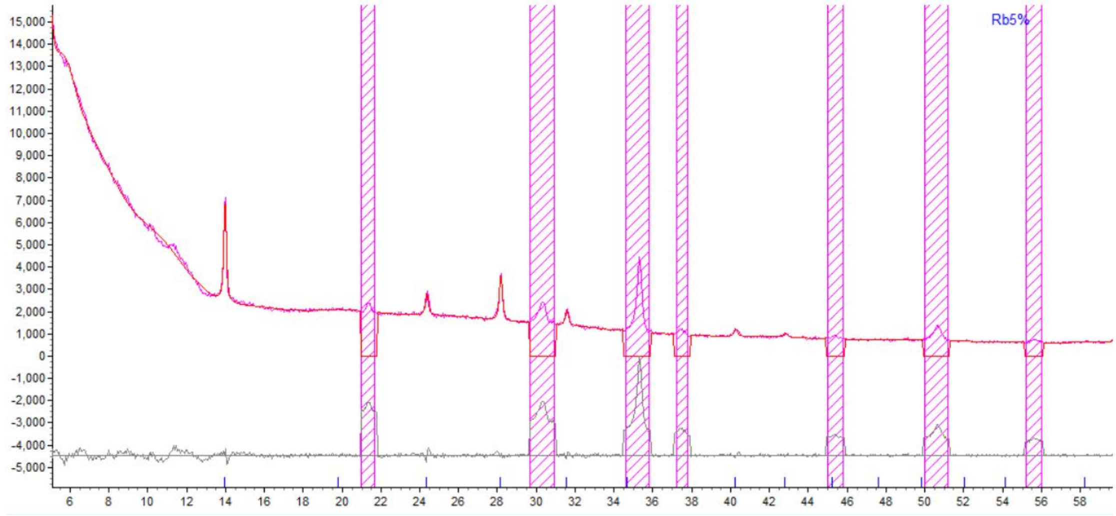

Figure S16. TOPAS fitting of the Rb5 sample.

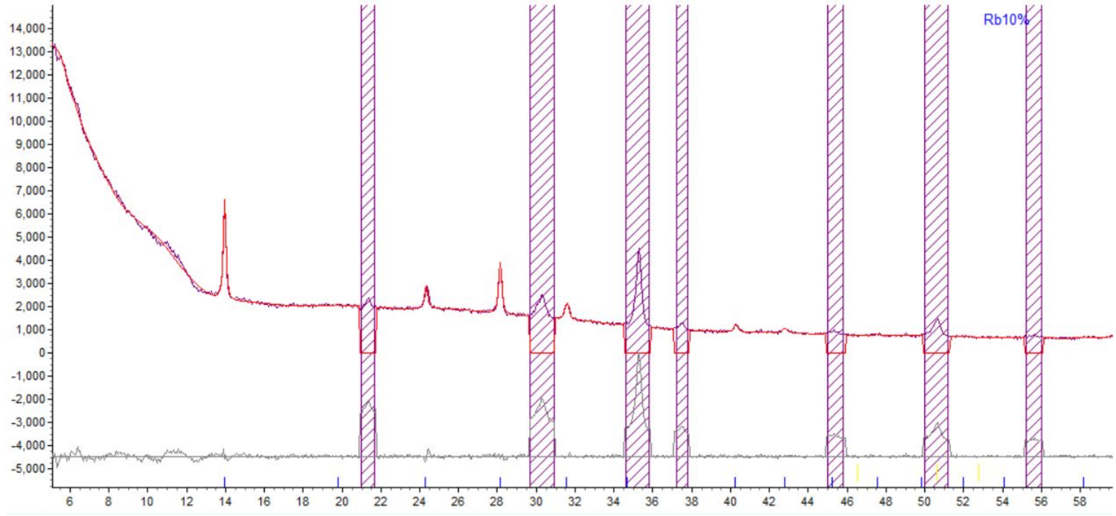

Figure S17. TOPAS fitting of the Rb10 sample.

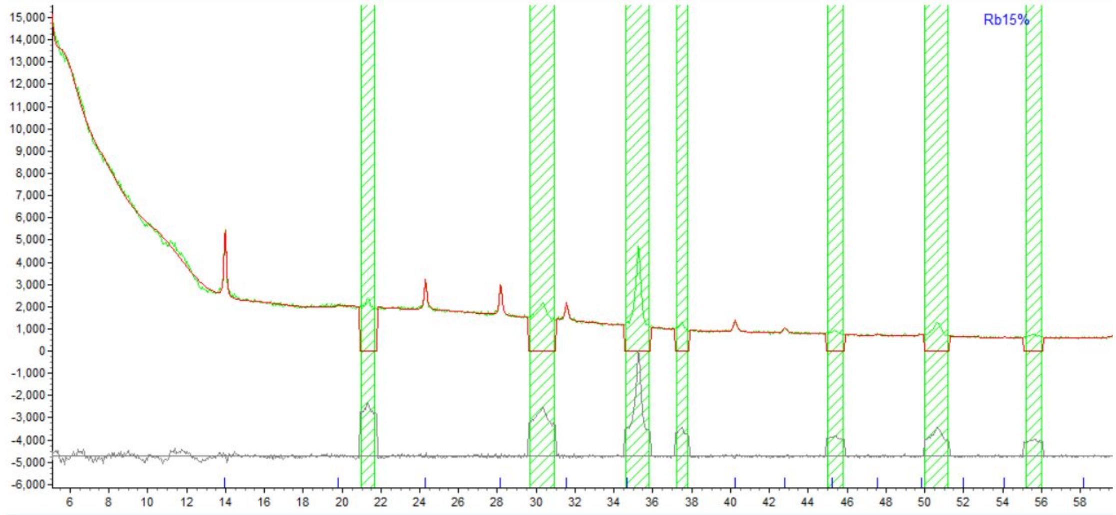

Figure S18. TOPAS fitting of the Rb15 sample.

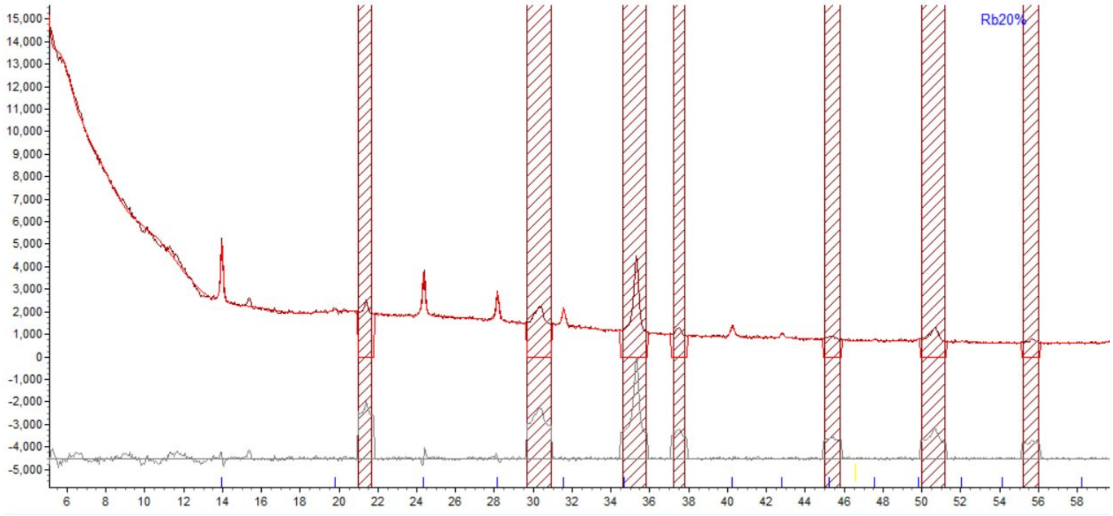

Figure S19. TOPAS fitting of the Rb20 sample.

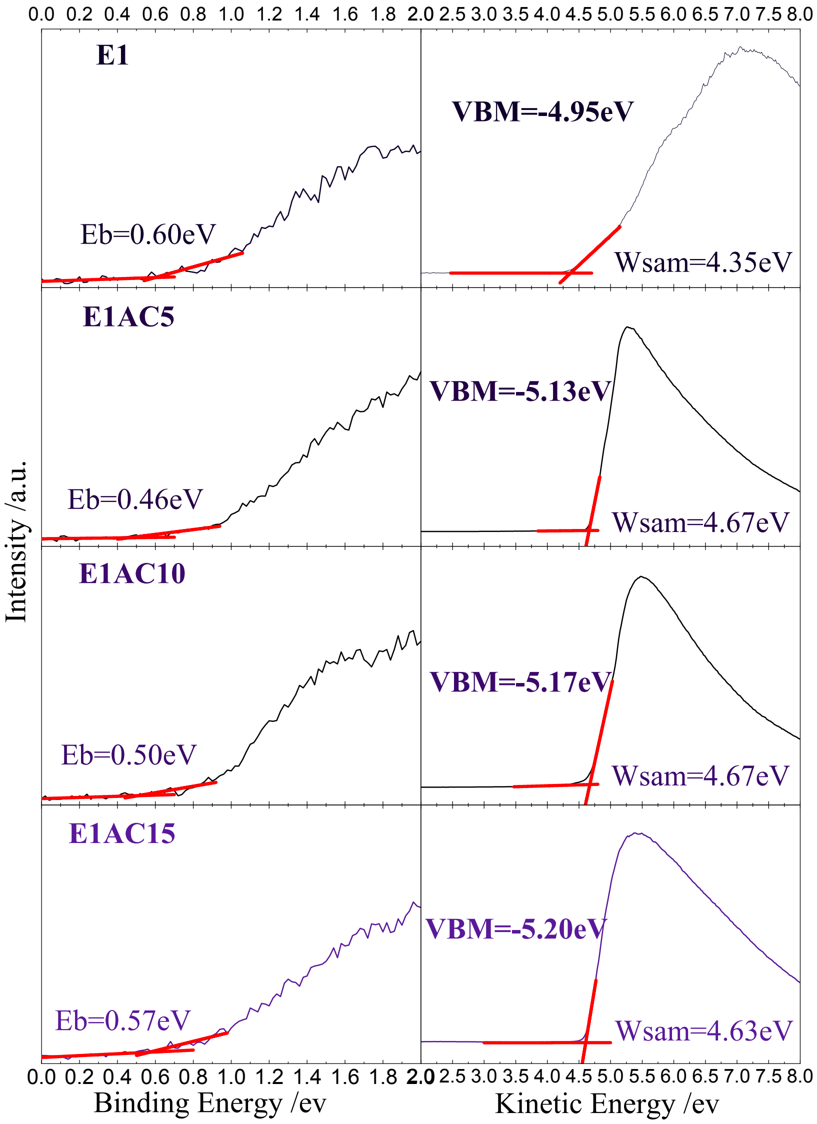

Figure S20. UPS raw data of the E1AC0-15 samples.

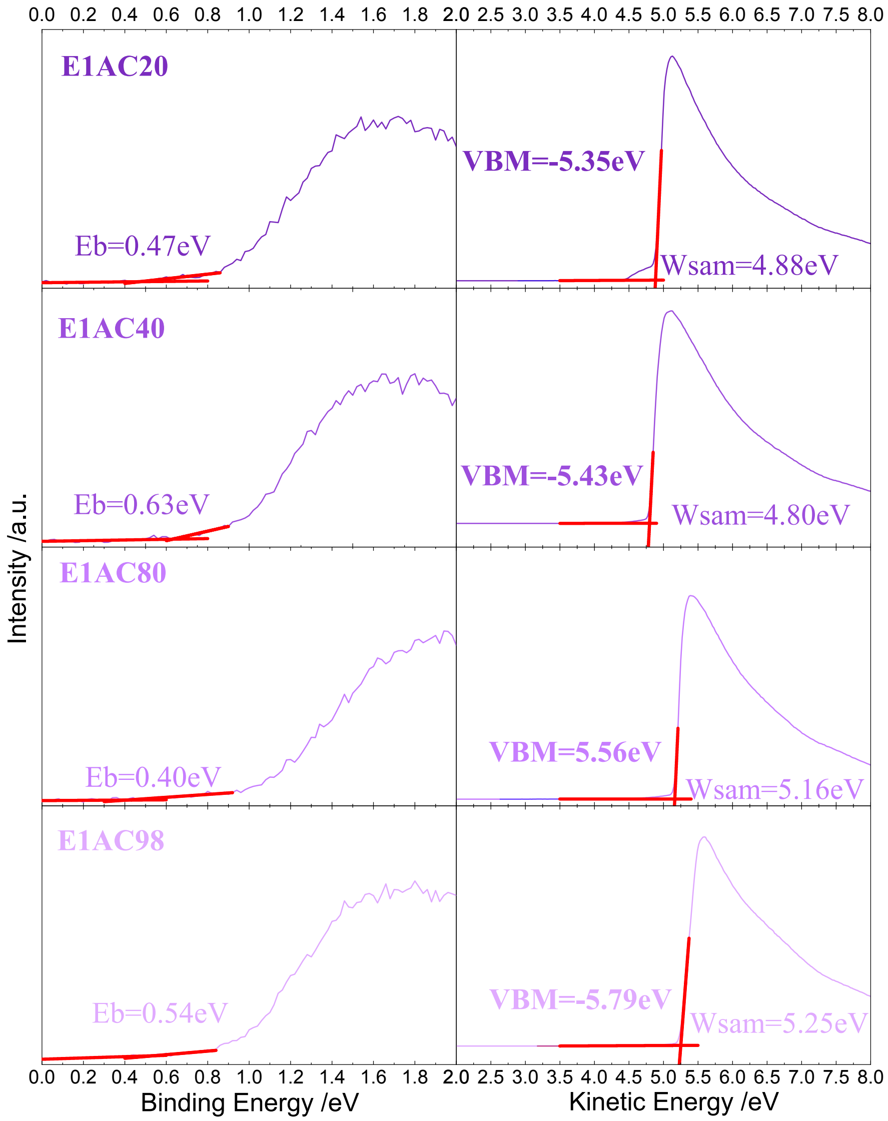

Figure S21. UPS raw data of the E1AC20-98 samples.

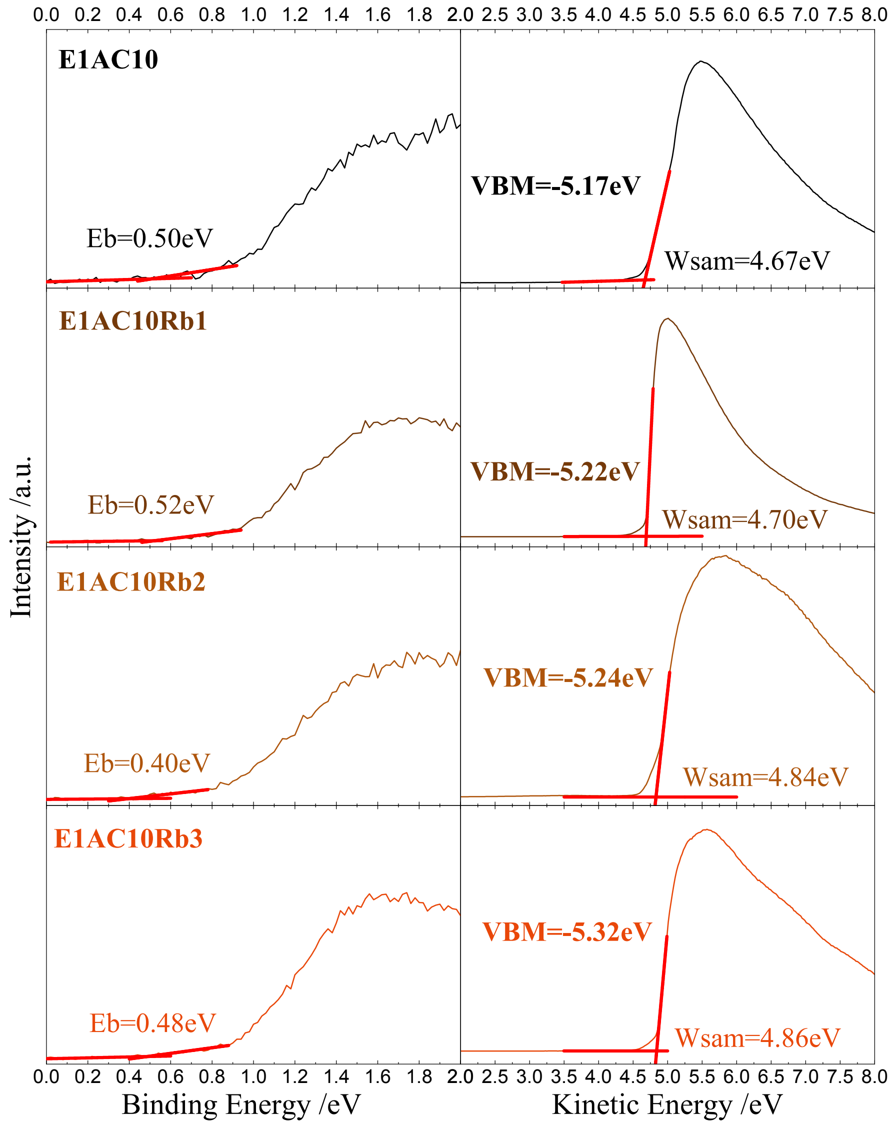

Figure S22. UPS raw data of the E1AC10Rb0-3 samples.

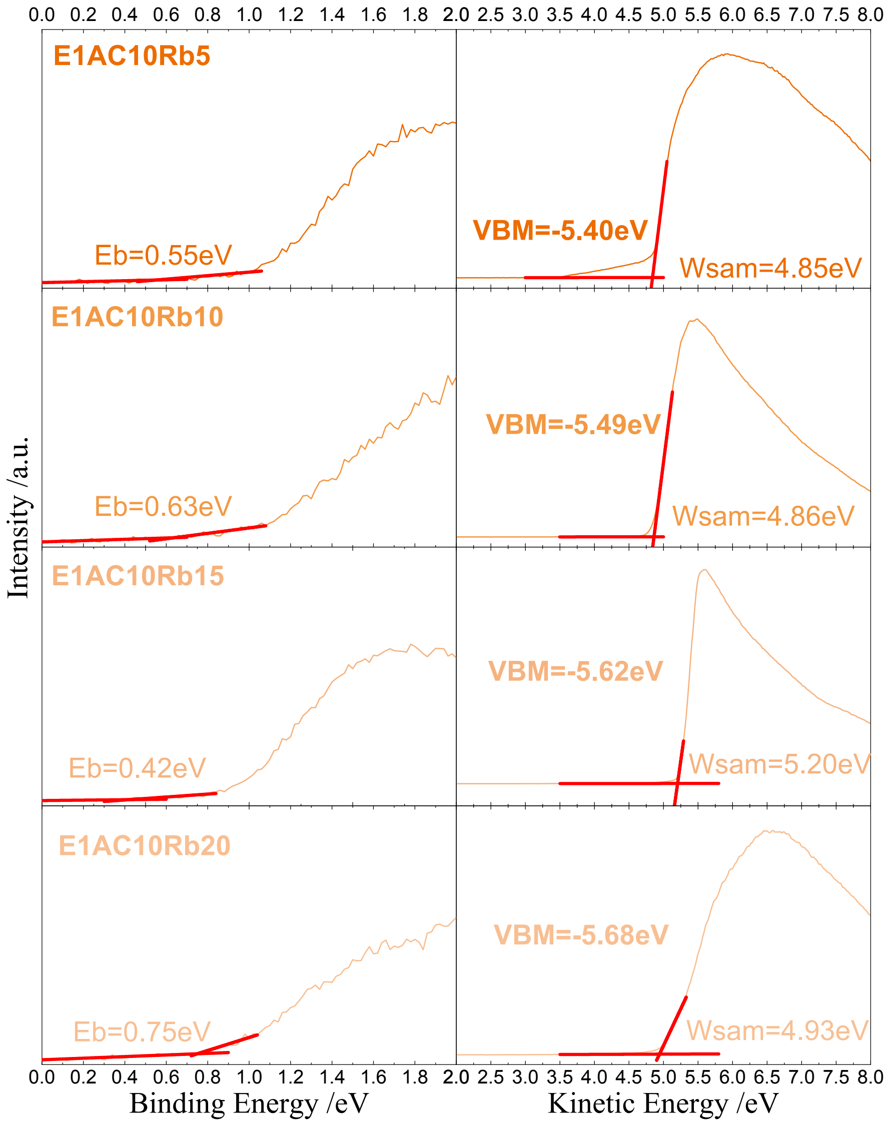

Figure S23. UPS raw data of the E1AC10Rb5-20 samples.

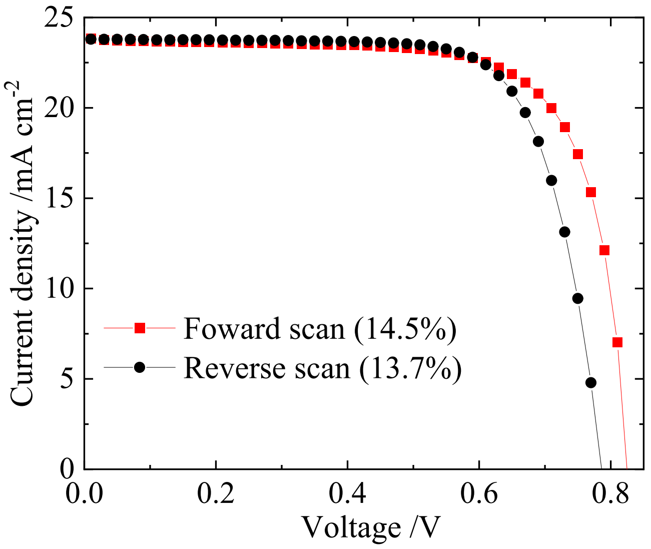

Figure S24. *J-V* scan curves showing the effect of hysteresis for the champion device.

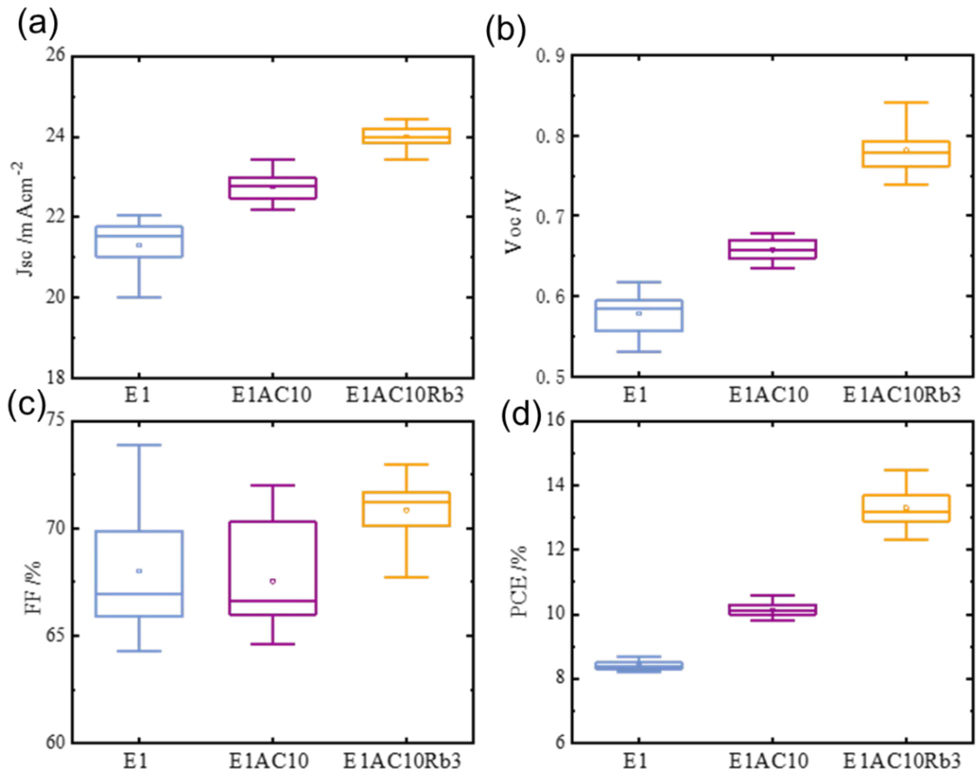

Figure S25. Boxplots of photovoltaic parameters for the devices made of E1, E1AC10 and E1AC10Rb3.

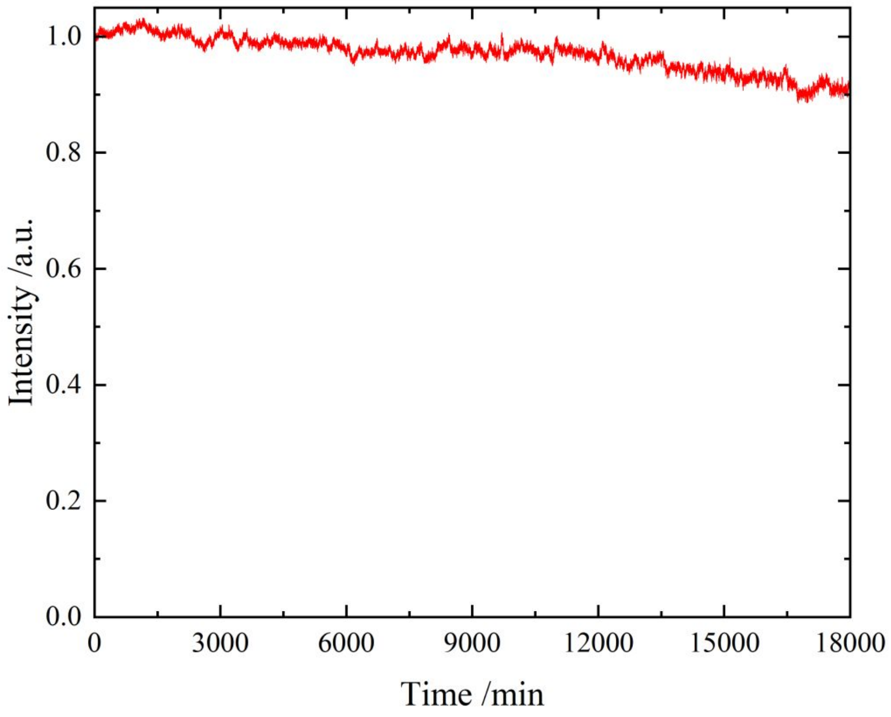

Figure S26. Device performance of the E1AC10Rb3 device measured under one-sun ambient air condition (relative humidity = 50%) at maximum-power point (MPP) for continuous irradiation up to 7.5 h.

Table S1. Sn^{2+} and Sn^{4+} proportions of E1, E1AC10 and E1AC10Rb3 obtained from XPS measurements.

**Sample**	**E1**	**E1AC10**	**E1AC10Rb3**
**Sn^{2+}**	68.4%	75.9%	97.2%
**Sn^{4+}**	31.6%	24.1%	2.8%

Table S2. Lattice parameters of the AC samples obtained from TOPAS simulations.

**Sample**	**Space group**	**a/Å**	**b/Å**	**c/Å**	**α=β=γ /deg**
**E1**	Pm-3m	6.3195	6.3195	6.3195	90
**E1AC5**	Pm-3m	6.3345	6.3345	6.3345	90
**E1AC10**	Pm-3m	6.3347	6.3347	6.3347	90
**E1AC15**	Pm-3m	6.3399	6.3399	6.3399	90
**E1AC20**	Pm-3m	6.3420	6.3420	6.3420	90
**E1AC40**	Pm-3m	6.3467	6.3467	6.3467	90

Table S3. Lattice parameters of the Rb samples obtained from TOPAS simulations.

**Sample**	**Space group**	**a/Å**	**b/Å**	**c/Å**	**α=β=γ /deg**
**E1AC10Rb1**	Pm-3m	6.3223	6.3223	6.3223	90
**E1AC10Rb2**	Pm-3m	6.3335	6.3335	6.3335	90
**E1AC10Rb3**	Pm-3m	6.3247	6.3247	6.3247	90
**E1AC10Rb5**	Pm-3m	6.3317	6.3317	6.3317	90
**E1AC10Rb10**	Pm-3m	6.3369	6.3369	6.3369	90
**E1AC10Rb15**	Pm-3m	6.3375	6.3375	6.3375	90
**E1AC10Rb20**	Pm-3m	6.3332	6.3332	6.3332	90

Table S4. Time coefficients (in ns) obtained from TCPSC measurements for the AC samples.

	E1	E1AC5	E1AC10	E1AC15	E1AC20	E1AC40	E1AC80
A1	0.853	0.776	0.73	0.789	0.846	0.442	0.643
A2	0.137	0.187	0.223	0.199	0.144	0.32	0.276
A3	0	0	0	0	0	0.242	0.081
τ1	6.07	6.96	7.3	5.84	5.44	1.29	0.03
τ2	17.3	18.3	17.88	16.97	10.43	1.45	0.53
τ3	0	0	0	0	0	3.46	3.47
ave τ	9.56	11.35	11.82	10.55	6.67	2.27	2.1

Table S5. Time coefficients (in ns) obtained from TCPSC measurements for the Rb samples.

	AC10	AC10 Rb1	AC10 Rb2	AC10 Rb3	AC10 Rb5	AC10 Rb10	AC10 Rb15	AC10 Rb20
A1	0.73	0.627	0.678	0.684	0.557	0.65	0.757	0.786
A2	0.223	0.299	0.283	0.268	0.389	0.321	0.23	0.157
τ1	7.3	9.22	10.12	12.43	10.63	8.27	7.48	7.39
τ2	17.88	21.73	28.11	30.36	26.11	22.38	20.27	18.47
ave τ	11.82	15.84	19.78	21.2	20.41	16.34	13.26	11.08

Table S6. Raw data of photovoltaic parameters for the E1 devices.

Device no.	Jsc /mA cm $^{-2}$	Voc /V	FF /%	PCE /%
1	20.02	0.552	72.2	8.0
2	20.09	0.572	68.8	7.9
3	21.14	0.581	64.7	7.9
4	20.59	0.586	66.9	8.1
5	21.02	0.592	66.8	8.3
6	22.04	0.583	66.1	8.5
7	21.25	0.583	67.6	8.4
8	20.72	0.589	67.1	8.2
9	21.07	0.590	66.6	8.3
10	21.71	0.592	65.6	8.4
11	21.43	0.595	66.2	8.4
12	21.93	0.587	66.5	8.6
13	21.60	0.589	67.0	8.5
14	21.78	0.598	66.2	8.6
15	21.76	0.598	65.9	8.6
16	21.84	0.601	65.8	8.7
17	21.66	0.611	65.3	8.7
18	21.71	0.610	64.4	8.5
19	21.63	0.617	65.5	8.7
20	21.68	0.615	64.3	8.6
21	21.13	0.554	71.1	8.3
22	21.92	0.557	68.2	8.3
23	20.30	0.554	72.8	8.2
24	20.36	0.578	69.9	8.2
25	20.39	0.584	69.7	8.3
26	21.95	0.531	72.0	8.4
27	21.93	0.532	71.6	8.3
28	21.44	0.532	73.9	8.4
29	21.50	0.574	68.7	8.5
30	21.56	0.532	72.7	8.3
mean±s.d.	21.31±0.61	0.579±0.025	68.0±2.8	8.4±0.2

Table S7. Raw data of photovoltaic parameters for the E1AC10 devices.

Device no.	Jsc /mA cm $^{-2}$	Voc /V	FF /%	PCE /%
1	22.43	0.670	66.6	10.0
2	22.28	0.672	66.2	9.9
3	22.18	0.671	66.6	9.9
4	22.99	0.677	64.6	10.0
5	22.79	0.674	65.8	10.1
6	22.72	0.643	70.7	10.3
7	22.53	0.635	71.3	10.2
8	22.76	0.647	70.3	10.4
9	22.33	0.640	72.0	10.3
10	23.08	0.653	66.0	10.0
11	22.77	0.651	66.9	9.9
12	22.76	0.655	65.9	9.8
13	23.34	0.647	70.4	10.6
14	22.99	0.641	71.7	10.6
15	22.20	0.656	67.4	9.8
16	22.34	0.663	66.7	9.9
17	22.68	0.662	66.5	10.0
18	22.91	0.666	66.5	10.5
19	22.47	0.670	66.2	10.0
20	22.86	0.652	67.2	10.0
21	22.98	0.656	65.8	9.9
22	22.72	0.653	67.2	10.0
23	23.40	0.662	65.1	10.1
24	23.02	0.660	66.8	10.1
25	23.45	0.661	66.2	10.3
26	23.04	0.661	67.0	10.2
27	22.95	0.678	64.9	10.1
28	22.76	0.673	65.9	10.1
29	22.74	0.645	70.3	10.3
30	22.37	0.642	71.5	10.3
mean±s.d.	22.76±0.34	0.658±0.012	67.5±2.3	10.1±0.2

Table S8. Raw data of photovoltaic parameters for the E1AC10Rb3 devices.

Device no.	Jsc /mA cm $^{-2}$	Voc /V	FF /%	PCE /%
1	23.71	0.843	68.6	13.8
2	24.17	0.793	68.8	13.2
3	23.46	0.771	69.7	12.6
4	23.96	0.781	70.7	13.2
5	24.43	0.785	72.8	14.0
6	23.45	0.751	69.7	12.3
7	24.21	0.838	70.4	14.3
8	23.92	0.788	71.5	13.5
9	24.13	0.775	72.7	13.6
10	24.37	0.752	71.6	13.1
11	23.96	0.818	71	13.9
12	24.23	0.783	71.7	13.6
13	23.76	0.756	69.1	12.4
14	24.09	0.749	70.5	12.7
15	23.89	0.763	71.9	13.1
16	23.91	0.841	72.1	14.5
17	24.32	0.785	70.1	13.4
18	23.74	0.761	72.3	13.1
19	24.10	0.760	67.7	12.4
20	23.94	0.783	72.3	13.6
21	24.21	0.775	68.0	12.8
22	23.81	0.739	70.7	12.4
23	24.05	0.761	71.3	13.1
24	24.22	0.793	73.0	14.0
25	24.33	0.780	71.7	13.6
26	23.94	0.777	71.2	13.2
27	24.05	0.770	71.3	13.2
28	23.80	0.829	71.3	14.1
29	23.84	0.806	71.3	13.7
30	24.13	0.759	70.6	12.9
mean±s.d.	24.00±0.25	0.782±0.028	70.9±1.4	13.3±0.6
