# Supplementary material for: Co-Cation Engineering via Mixing of Acetamidinium and Rubidium in FASnI3 for Tin Perovskite Solar Cells to Attain 14.5% Efficiency
Source: J Phys Chem Lett. 2024 Jul 24;15(30):7763–9. doi: 10.1021/acs.jpclett.4c01695 (PMC11299185; doi:10.1021/acs.jpclett.4c01695)
Supplement: Supplementary file 2 — jz4c01695_si_002.pdf [file jz4c01695_si_002.pdf]

jz-2024-01695j.R1

Name: Peer Review Information for "Co-Cation Engineering via Mixing of Acetamidinium and Rubidium in FASnI₃ for Tin Perovskite Solar Cells to Attain Efficiency 14.5%"

First Round of Reviewer Comments

Reviewer: 1

Comments to the Author

Diau et al. have presented an investigation into the enhanced efficiency of tin-based perovskite solar cells (TPSCs) by integrating co-cations acetamidinium (AC) and rubidium (Rb), achieving a reported efficiency of 14.5%. This performance is comparable to the highest efficiencies documented for TPSCs. The authors have employed a comprehensive array of analytical methodologies to substantiate their conclusions. Despite the strengths of this study, I believe further validation and discussion are necessary to fully support the authors' assertions.

The motivation for using co-cation doping to enhance TPSC performance was attributed partly to adjusting the tolerance factor. However, this explanation does not fully clarify why the specific combination of AC and Rb was chosen. Given the myriad possibilities for enhancing TPSC stability, the selection of AC and Rb suggests a particular rationale that the manuscript does not currently elucidate. Could the authors provide a more detailed justification for choosing this specific cation combination?

The TOF-SIMS data suggest segregation of FA and Rb, which appears to contradict the claim that these perovskites are A-site co-doped materials. This observation raises questions about the material's homogeneity and the efficacy of the doping strategy. Can the authors clarify why FA and Rb preferentially localize at the interfaces and surfaces, respectively, and discuss how this distribution aligns with their hypotheses about material improvements?

Given the relatively small ionic radius of Rb, its incorporation into the A-sites of the perovskite structure is intriguing. Can the authors provide explicit evidence demonstrating that Rb occupies A-site positions within the perovskite lattice? Additionally, if Rb is indeed positioned at A-sites, why does this not result in significant shifts in XRD peaks and absorption spectra, which would likely reflect changes in lattice parameters or electronic structure?

Lastly, I have wondered what the potential avenues are for further improving the PCE beyond 14.5% using the triple cation strategy?

Reviewer: 2

Comments to the Author

In this work, authors regulate the composition of tin based perovskite materials for lead free perovskite solar cells, and a best PCE of 14.5% was obtained. The following problems should be addressed.

(1) As incorporating Rb cations significantly increases the Voc of devices, the effect of Pb cations on the E1 materials and FASnI3 materials without AC cations should be studied.

(2) The full name of SA should be provided.

(3) Most of devices with high Voc use the electron transport material of ICBA, the performance of devices with ICBA instead of C60 should be examined.

(4) As shown in Table S8, the reproducibility of E1ACRb3 devices is poor. This problem should be addressed.

(5) The mechanism of high crystallinity in E1ACRb3 should be further studied.

Reviewer: 3

Comments to the Author

In this manuscript, Diau et al. included both acetamidinium and rubidium in varying proportions into the FASnI3 structure. It was discovered that adding 10% AC and 3% Rb optimizes the device, achieving a power conversion efficiency of 14.5% along with excellent shelf- and light-soaking stability. This is an interesting work and the manuscript is well organized. I would like to suggest its acceptance after the following comments were addressed.

1. It seems that the inclusion of Rb is critical to improve the device performance. How about improve the amount of Rb further in the film? How about adding Rb in the control sample without Ac cations?

2. In fig. 4e and 4f, the decay time of the sample with Ac is shorter than the control. How to explain this phenomenon?

3. The size of Rb is much smaller than other two cations, is there any shift of the XRD peak? If there is no shift of the peak, how to explain it?

4. In Figure 2 for the GIWAXS measurement, the diffraction peak assigned to δ-phase is not correct. Authors need to double check this peak.

5. In Figure 5c, can authors give the equivalent circuit of device?

6. Quasi-2D structure is generally used in high efficiency tin perovskite solar cells. Maybe it could be noted in the introduction part.

Author's Response to Peer Review Comments:

Editorial comments

1. Please include annotated version(s) of your revised publication file(s) with colored text or highlights indicating the revisions that you have made, and upload them as "Supporting Information for Review Only." Please also upload "clean" copies for publication. (No pphighlighting, annotations, or colored text permitted.)
Ans: We have provided both files as suggested.

2. Title: In both the main manuscript file and the Supporting Information, set the title in title case, with the first letter of each principal word capitalized.
Ans: We have changed the title with the first letter capitalized.

3. Headers: Remove the section heading(s) throughout the body of the manuscript (you can leave Methods, Abstract, and TOC Graphic headings).
Ans: The headers were removed as suggested.

4. TOC Graphic: Provide a TOC image per journal guidelines (2 in x 2 in; on the same page as the abstract) with the heading "TOC Graphic" above the graphic. The graphic should be in the form of a structure, graph, drawing, photograph, or scheme—or a combination. Non-scientific cartoon-like images or caricatures are discouraged. https://pubsapp.acs.org/paragonplus/submission/toc_abstract_graphics_guidelines.pdf
Ans: The TOC graphic is provided after the Abstract section.

5. Supporting Information Statement: A brief, nonsentence description of the actual contents of each supporting information file is required. This description should be labeled Supporting Information and should appear before the Acknowledgement and Reference sections. Examples of sufficient and insufficient descriptions are as follows: *Examples of sufficient descriptions: "Supporting Information: 1H NMR spectra for all compounds" or "Additional experimental details, materials, and methods, including photographs of experimental setup". *Examples of insufficient descriptions: "Supporting Information: Figures S1-S3" or "Additional figures as mentioned in the text".
Ans: The Supporting Information Statement is provided as suggested before Acknowledgement section highlighted in yellow.

6. References: In both the main file and the supporting information, fix the style of all references to use JPCL formatting (check all references carefully). ***JPC Letters reference formatting requires that journal references should contain: () around numbers; author names; article title (titles entirely in title case or entirely in lower case); abbreviated journal title (italicized); year (bolded); volume (italicized); and pages (first-last). Book references should contain author names; book title (in the same pattern);

publisher; city; and year. Websites must include date of access.
7. Supporting Information: Please number SI pages in the following format: "S1, S2…"
Ans: We have made modifications for the Reference section as suggested.

Reviewer(s)' Comments to Author:

Reviewer: 1

Recommendation: This paper may be publishable, but major revision is needed; I would like to be invited to review any future revision.

Comments:
Diau et al. have presented an investigation into the enhanced efficiency of tin-based perovskite solar cells (TPSCs) by integrating co-cations acetamidinium (AC) and rubidium (Rb), achieving a reported efficiency of 14.5%. This performance is comparable to the highest efficiencies documented for TPSCs. The authors have employed a comprehensive array of analytical methodologies to substantiate their conclusions. Despite the strengths of this study, I believe further validation and discussion are necessary to fully support the authors' assertions.
Ans: We thank the reviewer for the positive comments and suggestions. More details are given in the manuscript and supporting information.

The motivation for using co-cation doping to enhance TPSC performance was attributed partly to adjusting the tolerance factor. However, this explanation does not fully clarify why the specific combination of AC and Rb was chosen. Given the myriad possibilities for enhancing TPSC stability, the selection of AC and Rb suggests a particular rationale that the manuscript does not currently elucidate. Could the authors provide a more detailed justification for choosing this specific cation combination?
Ans: We thank the reviewer for raising this valuable comment. Incorporation of AC has the following reasons. The restricted C−N bond rotation in AC is due to the delocalized π-electron cloud over the N−C−N bond, which strengthens the resulting N−H···I bond, increasing the electrostatic interaction between the AC cation and the SnI_6^{4-} octahedron. These interactions can stabilize the perovskite matrix, thereby enhancing its stability (*ACS Appl. Mater. Interfaces* 2020, 12, 12, 13982–13987). For incorporation of Rb cation, it leads to higher-quality perovskite films with improved crystallinity and fewer defects. Due to the rapid crystallization rate of tin perovskite, pinholes easily form at the bottom of the perovskite layer. As demonstrated by TOF-SIMS in the manuscript, rubidium can effectively modify this bottom layer, providing a buried interface that passivates the TPSC. This enhancement is crucial for efficient charge transport and overall device performance. To make it clear, a few sentences were added in pages 4-5 highlighted in yellow and the above reference was cited.

The TOF-SIMS data suggest segregation of FA and Rb, which appears to contradict the claim that these perovskites are A-site co-doped materials. This observation raises questions about the material's homogeneity and the efficacy of the doping strategy. Can the authors clarify why FA and Rb preferentially localize at the interfaces and surfaces, respectively, and discuss how this distribution aligns with their hypotheses about material improvements?

Ans: We thank the reviewer for raising this valuable comment. FA constitutes the majority (87%) of the A-site cations in the tin perovskite structure. Consequently, the A-site is predominantly occupied by the FA ions. For 10% of AC, it mainly co-occupies with FA inside tin perovskite layer but with the tendency to passivate the surface where the FA vacancy defect exists. For only 3% of Rb, due to its smaller ionic radius, it can readily migrate through the defect channels within the perovskite lattice to reach and fill deeper defects. This process effectively passivates the interfacial defects at the bottom of the tin perovskite layer, leading to enhanced device performance. For the above reason, we chose AC and Rb as co-cations to passivate the tin perovskite defects.

Given the relatively small ionic radius of Rb, its incorporation into the A-sites of the perovskite structure is intriguing. Can the authors provide explicit evidence demonstrating that Rb occupies A-site positions within the perovskite lattice? Additionally, if Rb is indeed positioned at A-sites, why does this not result in significant shifts in XRD peaks and absorption spectra, which would likely reflect changes in lattice parameters or electronic structure?

Ans:

We thank the reviewer for raising this valuable comment. We conducted TOPAS fitting of the XRD data and the results are presented in the Supporting Information section. Based on the TOPAS analysis, we observed that doping with 3% Rb, compared to E1AC10, resulted in lattice shrinkage, which caused a slight blue shift in the PL spectrum, indicating that a small amount of Rb was incorporated into the lattice. However, the ionic radius of Rb is smaller than those of AC and FA, making it difficult for Rb to occupy the A-site of the perovskite lattice in large quantities. Instead, Rb primarily fills the A-site defects formed during the perovskite crystallization process, effectively passivating the defects in the bottom of the perovskite thin films.

Lastly, I have wondered what the potential avenues are for further improving the PCE beyond 14.5% using the triple cation strategy?

Ans:

We thank the reviewer for raising this valuable comment. There is an opportunity to improve efficiency by designing and developing new electron transport layers that enhance electron mobility and stability, consequently, increase overall efficiency. This work is underway in our laboratory.

Reviewer: 2

Recommendation: This paper is probably publishable, but major revision is needed; I do not need to see future revisions.

Comments:
In this work, authors regulate the composition of tin based perovskite materials for lead free perovskite solar cells, and a best PCE of 14.5% was obtained. The following problems should be addressed.
(1) As incorporating Rb cations significantly increases the Voc of devices, the effect of Pb cations on the E1 materials and $FASnI_3$ materials without AC cations should be studied.
Ans: We thank the reviewer for raising this valuable comment. The results of adding Rb to the control sample without AC cations are shown below. 3% of Rb was added into E1 without AC, the PCE was 10.1%. When the Rb content is increased to 5%, the device performance declines. The optimal Rb composition is 3%. Without AC, the results indicate that 3% Rb effectively passivates the bottom of E1, thereby improving performance.

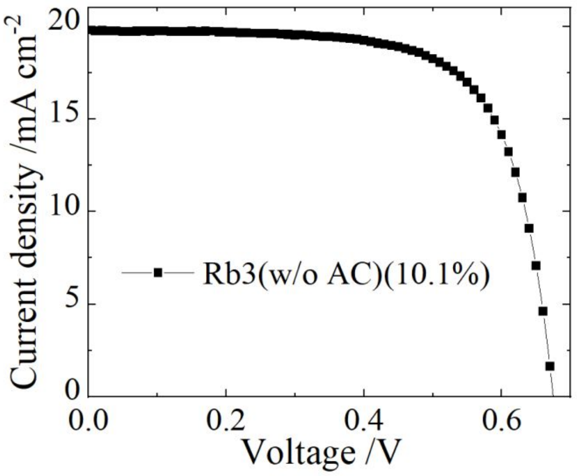

(2) The full name of SA should be provided.
Ans: We thank the reviewer to give this suggestion. SA stands for sulfamic acid. We have implemented the suggestion on page 1 of the revised supporting information highlighted in yellow.

(3) Most of devices with high Voc use the electron transport material of ICBA, the performance of devices with ICBA instead of C60 should be examined.
Ans: We thank the reviewer for raising this valuable comment. We have tried experiment of ICBA instead of C60, but the performance of ICBA-based is poor. PCE is

2.1% as the results shown below. The ICBA device was quite unstable so that the device performance should be measured inside the glovebox. Currently all the devices were measured in the ambient air condition without encapsulation. For the above reason, We could not obtain good performance for the ICBA devices.

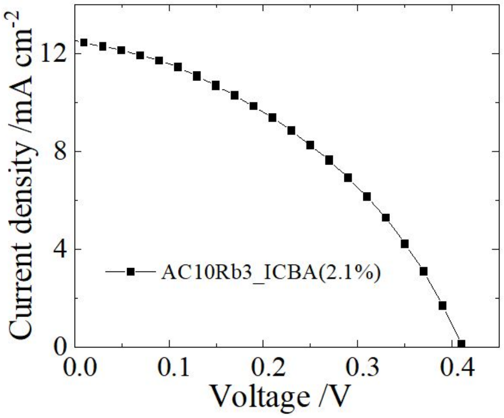

(4) As shown in Table S8, the reproducibility of E1ACRb3 devices is poor. This problem should be addressed.

Ans:

We thank the reviewer for raising this valuable comment. We have repeated the performance experiments with total 30 devices. As a results, the statistical data shown in Figure S25 has been updated. The performance parameters for each device are shown in Tables S6-S8.

(5) The mechanism of high crystallinity in E1ACRb3 should be further studied.

Ans:

We thank the reviewer for raising this valuable comment. TOF-SIMS analysis reveals that AC and Rb effectively passivated the surface and bottom defects of tin perovskite, leading to a significant improvement in its crystallinity. Additionally, XRD results demonstrated that the crystalline orientation of E1AC10Rb3 was enhanced compared to the others.

Reviewer: 3

Recommendation: This paper is publishable subject to minor revisions noted. Further review is not needed.

Comments:

In this manuscript, Diau et al. included both acetamidinium and rubidium in varying

proportions into the FASnI3 structure. It was discovered that adding 10% AC and 3% Rb optimizes the device, achieving a power conversion efficiency of 14.5% along with excellent shelf- and light-soaking stability. This is an interesting work and the manuscript is well organized. I would like to suggest its acceptance after the following comments were addressed.

1. It seems that the inclusion of Rb is critical to improve the device performance. How about improve the amount of Rb further in the film? How about adding Rb in the control sample without Ac cations?

Ans:

We thank the reviewer for raising this valuable comment. The results of adding Rb to the control sample without AC cations are shown below. 3% of Rb was added into E1 without AC, the PCE was 10.1%. When the Rb content is increased to 5%, the device performance declines. The optimal Rb composition is 3%. Without AC, the results indicate that 3% Rb effectively passivates the bottom of E1, thereby also improving the device performance.

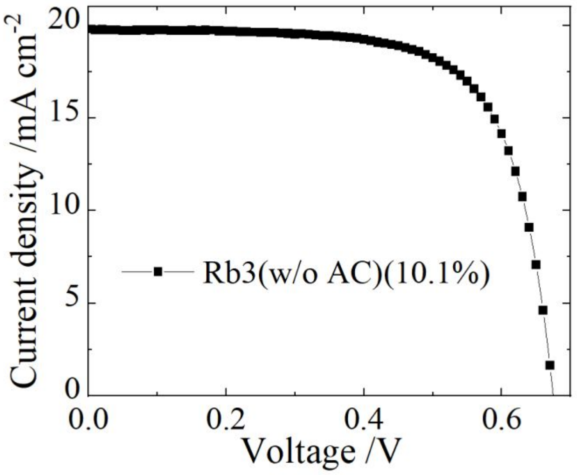

2. In fig. 4e and 4f, the decay time of the sample with Ac is shorter than the control. How to explain this phenomenon?

Ans:

We thank the reviewer to raise this question. The shorter picosecond decay of the AC sample compared to the pristine sample can be ascribed to differences in the defect density between these samples. The picosecond decay components were often associated with the trap state-mediated recombination processes. However, the differences in the time constants are not substantial, considering the signal-to-noise ratio in the picosecond region. That is why we haven't emphasized the picosecond decay dynamics in our manuscript due to their large uncertainties. The slowdown of thermalization and retarded charge recombination (the picosecond component was

unobservable) of the Rb sample are the most prominent observations for this study, which were clarified by the fs TAS results shown in Figures 4e and 4f.

3. The size of Rb is much smaller than other two cations, is there any shift of the XRD peak? If there is no shift of the peak, how to explain it?

Ans:

We thank the reviewer for raising this question. It is true that the XRD spectra show almost no deviation for these samples, which is attributed to the small particle size of Rb. This small size allows Rb to primarily modify A-site defects and crystal defects in the perovskite. To explain this phenomenon, we conducted TOPAS fitting of the XRD data and the results are presented in the Supporting Information section. Based on the TOPAS analysis, we observed that doping with 3% Rb, compared to E1AC10, resulted in lattice shrinkage, which caused a slight blue shift in the PL spectrum, indicating that a small amount of Rb was incorporated into the lattice.

4. In Figure 2 for the GIWAXS measurement, the diffraction peak assigned to δ-phase is not correct. Authors need to double check this peak.

Ans: We thank the reviewer for raising this valuable comment. We measured the XRD spectrum of SnI_2 as shown below. Upon calculation, we found that the peak initially identified as δ-phase should correspond to the SnI_2 species. Consequently, we corrected the GIWAXS spectrum, indicating that this peak should be attributed to SnI_2 rather than the δ-phase (*Adv. Energy Mater.*, *13*(27), 2300760.). For this reason, the labels in Figures 2j-l have been corrected.

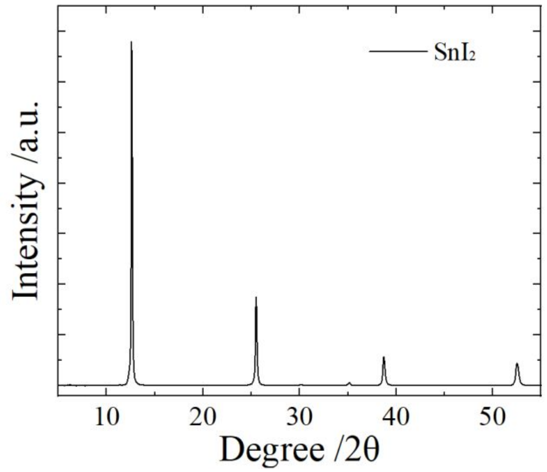

5. In Figure 5c, can authors give the equivalent circuit of device?

Ans: We thank the reviewer for raising this suggestion. Accordingly, we have added the equivalent circuit as an inset in Figure 5c of the revised manuscript.

6. Quasi-2D structure is generally used in high efficiency tin perovskite solar cells. Maybe it could be noted in the introduction part.

Ans: We thank the reviewer for give this suggestion. Accordingly, we have added a few sentences to highlight the importance of the 2D/3D structures in TPSC on page 3 of the revised manuscript highlighted in yellow. Two references (9, 10) were also cited to support our statements.
